# Higher thyrotropin leads to unfavorable lipid profile and somewhat higher cardiovascular disease risk: evidence from multi-cohort Mendelian randomization and metabolomic profiling

**DOI:** 10.1186/s12916-021-02130-1

**Published:** 2021-11-03

**Authors:** Nicolien A. van Vliet, Maxime M. Bos, Carisha S. Thesing, Layal Chaker, Maik Pietzner, Evelyn Houtman, Matt J. Neville, Ruifang Li-Gao, Stella Trompet, Rima Mustafa, Fariba Ahmadizar, Marian Beekman, Mariska Bot, Kathrin Budde, Constantinos Christodoulides, Abbas Dehghan, Christian Delles, Paul Elliott, Marina Evangelou, He Gao, Mohsen Ghanbari, Antonius E. van Herwaarden, M. Arfan Ikram, Martin Jaeger, J. Wouter Jukema, Ibrahim Karaman, Fredrik Karpe, Margreet Kloppenburg, Jennifer M. T. A. Meessen, Ingrid Meulenbelt, Yuri Milaneschi, Simon P. Mooijaart, Dennis O. Mook-Kanamori, Mihai G. Netea, Romana T. Netea-Maier, Robin P. Peeters, Brenda W. J. H. Penninx, Naveed Sattar, P. Eline Slagboom, H. Eka D. Suchiman, Henry Völzke, Ko Willems van Dijk, Raymond Noordam, Diana van Heemst, BBMRI Metabolomics Consortium

**Affiliations:** 1 Department of Internal Medicine, Section of Gerontology and Geriatrics, Leiden University Medical Center, PO Box 9600, 2300 RC Leiden, The Netherlands; 2 Department of Epidemiology, Erasmus MC, Rotterdam, The Netherlands; 3 Amsterdam UMC, Vrije Universiteit, Department of Psychiatry, Amsterdam Public Health research institute, Amsterdam, The Netherlands; 4 Academic Center for Thyroid Diseases, Erasmus MC, Rotterdam, The Netherlands; 5 Department of Internal Medicine, Erasmus MC, Rotterdam, The Netherlands; 6 Computational Medicine, Berlin Institute of Health (BIH), Charité-Universitätsmedizin Berlin, Berlin, Germany; 7 Department of Biomedical Data Sciences, section of Molecular Epidemiology, Leiden University Medical Center, Leiden, The Netherlands; 8 NIHR Oxford Biomedical Research Centre, Oxford University Hospitals Foundation Trust, Oxford, UK; 9 Radcliffe Department of Medicine, Oxford Centre for Diabetes, Endocrinology, and Metabolism, University of Oxford, Oxford, UK; 10 Department of Clinical Epidemiology, Leiden University Medical Center, Leiden, The Netherlands; 11 MRC Centre for Environment and Health, Department of Epidemiology and Biostatistics, School of Public Health, Imperial College London, London, UK; 12 Institute of Clinical Chemistry and Laboratory Medicine, University Medicine Greifswald, Greifswald, Germany; 13 Dementia Research Institute at Imperial College London, London, UK; 14 Institute of Cardiovascular and Medical Sciences, College of Medical, Veterinary and Life Sciences, University of Glasgow, Glasgow, UK; 15 NIHR Biomedical Research Centre, Imperial College London, London, UK; 16 BHF Imperial College Centre for Research Excellence, Imperial College London, London, UK; 17 Department of Mathematics, Faculty of Natural Sciences, Imperial College London, London, UK; 18 Department of Laboratory Medicine, Radboud Laboratory for Diagnostics (RLD), Radboud University Medical Center, Nijmegen, The Netherlands; 19 Department of Internal Medicine, Division of Endocrinology, Radboud University Medical Center, Nijmegen, The Netherlands; 20 Department of Internal Medicine and Radboud Center for Infectious Diseases, Radboud University Medical Center, Nijmegen, The Netherlands; 21 Department of Cardiology, Leiden University Medical Center, Leiden, The Netherlands; 22 Netherlands Heart Institute, Utrecht, The Netherlands; 23 Department of Rheumatology, Leiden University Medical Center, Leiden, The Netherlands; 24 Department of Orthopaedics, Leiden University Medical Center, Leiden, The Netherlands; 25 Institute for Evidence-Based Medicine in Old Age (IEMO), Leiden, The Netherlands; 26 Department of Public Health and Primary Care, Leiden University Medical Center, Leiden, The Netherlands; 27 BHF Glasgow Cardiovascular Research Centre, Faculty of Medicine, Glasgow, UK; 28 Max Planck Institute for Biology of Ageing, Cologne, Germany; 29 Institute for Community Medicine, University Medicine Greifswald, Greifswald, Germany; 30 Department of Human Genetics, Leiden University Medical Center, Leiden, The Netherlands; 31 Department of Internal Medicine, Division of Endocrinology, Leiden University Medical Center, Leiden, The Netherlands; 32 Einthoven Laboratory for Experimental Vascular Medicine, Leiden University Medical Center, Leiden, The Netherlands

**Keywords:** Thyroid hormones, Coronary artery disease, Metabolomics, Mendelian randomization

## Abstract

**Background:**

Observational studies suggest interconnections between thyroid status, metabolism, and risk of coronary artery disease (CAD), but causality remains to be proven. The present study aimed to investigate the potential causal relationship between thyroid status and cardiovascular disease and to characterize the metabolomic profile associated with thyroid status.

**Methods:**

Multi-cohort two-sample Mendelian randomization (MR) was performed utilizing genome-wide significant variants as instruments for standardized thyrotropin (TSH) and free thyroxine (fT4) within the reference range. Associations between TSH and fT4 and metabolic profile were investigated in a two-stage manner: associations between TSH and fT4 and the full panel of 161 metabolomic markers were first assessed hypothesis-free, then directional consistency was assessed through Mendelian randomization, another metabolic profile platform, and in individuals with biochemically defined thyroid dysfunction.

**Results:**

Circulating TSH was associated with 52/161 metabolomic markers, and fT4 levels were associated with 21/161 metabolomic markers among 9432 euthyroid individuals (median age varied from 23.0 to 75.4 years, 54.5% women). Positive associations between circulating TSH levels and concentrations of very low-density lipoprotein subclasses and components, triglycerides, and triglyceride content of lipoproteins were directionally consistent across the multivariable regression, MR, metabolomic platforms, and for individuals with hypo- and hyperthyroidism. Associations with fT4 levels inversely reflected those observed with TSH. Among 91,810 CAD cases and 656,091 controls of European ancestry, per 1-SD increase of genetically determined TSH concentration risk of CAD increased slightly, but not significantly, with an OR of 1.03 (95% CI 0.99–1.07; *p* value 0.16), whereas higher genetically determined fT4 levels were not associated with CAD risk (OR 1.00 per SD increase of fT4; 95% CI 0.96–1.04; *p* value 0.59).

**Conclusions:**

Lower thyroid status leads to an unfavorable lipid profile and a somewhat increased cardiovascular disease risk.

**Supplementary Information:**

The online version contains supplementary material available at 10.1186/s12916-021-02130-1.

## Background

Hypothyroidism, defined by high thyroid stimulating hormone (TSH) and low free thyroxine (fT4) levels, and subclinical hypothyroidism, defined by high TSH and fT4 within the reference range, are associated with higher total cholesterol, low-density lipoprotein cholesterol (LDL-c), and triglyceride levels [1, 2], and subclinical hypothyroidism has been associated with higher coronary artery disease (CAD) risk [3]. However, two recent randomized placebo-controlled trials on levothyroxine treatment in older adults with subclinical hypothyroidism did not find a reduction in cardiovascular events [4, 5], possibly due to a lack of statistical power [6].

Mendelian randomization (MR) studies [7] and studies using metabolomics data can further elaborate on the possible causal role of thyroid status in CAD [8]. Previous MR studies on thyroid status and CAD were performed in multi-ancestry populations [9–12], while thyroid function [13], prevalence of thyroid dysfunction [13, 14], and risk of myocardial infarction [15] all vary by ancestry. Moreover, genetic variants for thyroid parameters were discovered in European-ancestry populations only [16]. We hypothesized that performing MR in an exclusively European sample could provide a more accurate effect estimation. In addition, metabolomic profiling can be used as intermediate phenotype, to investigate early subclinical stages of diseases, especially when considering the lipoprotein subclasses and their contents [17, 18]. Recently, findings from a Brazilian cohort showed already promising results showing subclinical thyroid function to be related to unfavorable lipid profile using a metabolomics platform [19, 20].

We aimed to investigate the potential causal role of thyroid status in cardiovascular disease by assessment of the association between TSH and fT4 levels and CAD using MR in European-ancestry cohorts. Additionally, we investigated the association between thyroid status and metabolomic profile in two stages. First, associations between TSH and fT4 concentrations within the reference range were tested for the complete panel of 161 metabolomic markers. Next, robustness of associations between TSH and fT4 and the metabolomic markers identified in stage one, was tested with MR and a different NMR-metabolomics platform. Since the multivariable-adjusted regression and MR analyses methods are sensitive to different sources of bias, residual confounding and unbalanced horizontal pleiotropy respectively, triangulation of evidence can contribute to causal inference of observational findings [21]. The consistency of associations with metabolomic markers was also examined in individuals with thyroid dysfunction.

## Methods

### Study populations for multivariable-adjusted regression analyses on the metabolomic profile

We strived to include as much cohorts as possible with data on exposure and outcome being measured in European-ancestry participants. In the end, data from six European-ancestry cohorts were used for first stage analysis of circulating metabolomic marker concentrations and thyroid status; the 500 Functional Genomics Study (500FG) (*n* = 421) [22], the Genetics, Arthrosis and Progression study (GARP) (*n* = 321) [23], the Leiden Longevity Study (LLS) (*n* = 486) [24], the Netherlands Study of Depression and Anxiety (NESDA) (*n* = 2906) [25], PROSPER (*n* = 5316) [26], and the Rotterdam Study (RS) (*n* = 1690) [27] (detailed description in Additional File 1: Supplementary Materials). We used data from Study of Health in Pomerania (SHIP) as validation (*n* = 983) using different metabolomic profiling methods [28]. Each participating study obtained written informed consent from all participants and approval from the appropriate local institutional review boards.

### Thyroid parameters for multivariable regression analyses

For the multivariable regression analyses, TSH and fT4 were measured according to a standardized protocol (See Additional File 1: supplementary Materials). For analyses on TSH and fT4 within the reference range, cohort-specific reference ranges were used after which TSH and fT4 levels were inverse normal transformed to approximate normal distribution and facilitate comparison between cohorts. Biochemical thyroid dysfunction was also based on cohort-specific reference ranges; overt hyperthyroidism was defined by TSH levels below the reference range and fT4 levels above the reference range, overt hypothyroidism was defined by either TSH > 20 mIU/L or TSH below 20 mIU/L but above the reference range and fT4 below the reference range.

### Genetic instruments for TSH and fT4

Genetic instruments for TSH and fT4 concentrations were extracted from the largest genome-wide association studies (GWAS) meta-analysis on thyroid function comprising 72,167 European-ancestry participants [16]. A total of 62 independent single nucleotide polymorphisms (SNPs) were identified for circulating TSH (GWAS-based 9.4% explained variance) and 31 independent SNPs for circulating fT4 (GWAS-based 4.8% explained variance) [16] (Additional File 1: Online Table 1). Median F-statistics was 54 (range 32 to 576) for the TSH instruments and 43 (range 30 to 394) for the fT4 instruments.

### Outcome sources for metabolomic profile

Data for MR analyses on thyroid status and metabolomic profile were derived from four sources; MAGNETIC consortium (*n* = 24,925; downloaded from: http://www.computationalmedicine.fi/data#NMR_ GWAS)) [29], the Oxford Biobank (*n* = 6616) [30], the Netherlands Epidemiology of Obesity Study (*n* = 4734) [31], and PROspective Study of Pravastatin in the Elderly at Risk (PROSPER) (*n* = 2343) [26] (Additional File 1: Supplementary Materials). Data of the MAGNETIC consortium was publicly available. For the other studies, linear regression analyses were performed between the SNPs and standardized metabolomic marker concentrations (mean = 0, SD = 1), adjusted for age, sex, and up to ten principal components. Findings were validated in the Airwave Health Monitoring Study (Airwave) (*n* = 2021) that used a different NMR platform [32].

### Metabolomic profile measurements for multivariable-adjusted regression and Mendelian randomization analyses

We used metabolomic profile measurements performed on a high-throughput proton NMR platform (Nightingale Health Ltd., Helsinki, Finland) [33]. This method provides quantification of lipoprotein subclass profiling with lipid concentrations within 14 subclasses, fatty acid composition and other small molecules including glycolysis-related metabolites, amino acids, and ketone bodies [33] (total 161 metabolomic markers). Out of the 161 metabolic markers, 116 were included in the GWAS of the MAGNETIC consortium. Metabolomic profiling for SHIP and Airwave was generated by Bruker IVDr LIpoprotein Subclass Analysis (B.I.-LISA; Bruker Biospin, Rheinstetten, Germany) [34–36]. Out of the 105 quantified lipoprotein subclasses, 57 subclasses overlapped with Nightingale. Methodological details are described in the Additional File 1: Supplementary Materials.

### Outcome sources for CAD

For MR analyses on thyroid status and CAD, we used data from three studies with European-ancestry participants; CARDIoGRAM consortium (22,233 cases and 64,762 controls; downloaded from: http://www.cardiogramplusc4d.org/data-downloads/) [37], UK Biobank (52,946 cases and 393,549 controls) [38], and FinnGen (16,631 cases and 197,780 controls; freeze 5; downloaded from: https://www.finngen.fi/en/access_results) [39] to perform MR analyses using maximum sample size and to examine consistency of the MR results across the different cohorts. Case definitions are described in the Additional File 1: Supplementary Materials.

### Statistical analyses

For analyses on circulating metabolomic marker concentrations, values were natural log-transformed and subsequently standardized for analyses.

For the multivariable regression analyses, a prespecified analysis plan and syntax were distributed among cohorts. Population characteristics were derived as number (percentage) for categorical variables, mean and SD for normally distributed variables and median, and interquartile range (IQR) for non-normally distributed variables. Multivariable linear regression analyses were performed locally, and summarized results were collected centrally for quality control and meta-analysis. The main analysis was adjusted for age, sex, body mass index (BMI), and smoking (current versus former or never), which were considered major confounders. Given potential heterogeneity among cohorts included in the multivariable regression analyses, the meta-analysis comprised a random-effects model according to restricted maximum likelihood (REML). Sensitivity analyses were done with participants excluded who used thyroid therapy or lipid-lowering medication (defined by ATC codes H03 and C10, respectively) or medication for treatment of diabetes mellitus (DM), reported a history of thyroid disease or DM, or had fasting glucose > 7 mmol/L. Results for the multivariable regression analyses are presented as the association between one SD higher TSH and fT4 levels within the reference range and metabolomic marker concentrations in SD with 95% CI. For associations of thyroid dysfunction, regressed differences in circulating metabolomic marker concentrations in SD are shown for overt hyper- and hypothyroid individuals compared to euthyroid individuals. Analyses in this stage were hypothesis-free, and therefore, Bonferroni correction was applied based on 37 uncorrelated metabolomic markers (as previously applied [18]), resulting in a two-sided *p* value threshold of less than 1.34 × 10^−3^ (0.05/37). Circos plots were used to summarize and visually compare the fT4 and TSH results. Circos plots were created using EpiViz (version 0.1.0, https://github.com/mattlee821/EpiViz/), a Shiny web application and R package built using R (version 3.6.2) and Shiny (version 1.4.0). EpiViz adapts and builds on the Circlize [40] and ComplexHeatmap [41] R packages to create Circos plots compatible with association analysis data.

### Mendelian randomization analyses

Two-sample MR analyses were conducted using summary-level data from relatively independent GWAS from exposure and outcomes [42]. We extracted the association of each genetic variant for TSH and fT4 from summary data of GWAS for circulating metabolomic markers and CAD. All palindromic SNPs, which are SNPs with an effect allele frequency close to 0.5 in combination with alleles that correspond to nucleotides that pair with each other, were excluded prior to analyses, as being default in the TwoSampleMR package. Furthermore, to prevent overestimation of the precision of the causal effects, we excluded all SNPs in linkage disequilibrium at *R*^2^ > 0.001 from analyses as well. From each of the remaining SNPs, we calculated the explained variance (as (*β* x √(2 × minor allele frequency × (1 − minor allele frequency)))^2^ × 100) and F-statistics (as (*β*/standard error)^2^).

Our main analyses were inverse variance-weighted (IVW) analyses, which provide a weighted mean estimate of the association of the genetically determined exposure and the outcome assuming none of the instruments were invalid using additive random effects [43]. We performed weighted median estimator (WME), MR Egger regression, and MR pleiotropy residual sum and outlier (MR-PRESSO) analyses as sensitivity analyses to take into account possible bias caused by directional pleiotropy [43, 44]. MR-Egger is similar to IVW but does not force the regression line (i.e., of the SNP-thyroid status trait association on the SNP-metabolomic measure association) through the intercept. MR-Egger is statistically less efficient (providing wider confidence intervals) but provides a causal estimate (i.e., the regression slope) that is corrected for directional horizontal pleiotropy and a non-zero intercept which is an indication of the existence of directional pleiotropy. The weighted-median estimator is valid if more than 50% of the weight of the genetic instrument is from valid variants (i.e., if one single SNP or several SNPs jointly contributing 50% or more of the weight in the MR analysis exhibit horizontal pleiotropy the calculated effect estimate may be biased). We first performed MR analyses on each dataset separately and subsequently meta-analyzed the summary estimates using fixed-effects models. Effect estimates for MR analyses with metabolomic profile represent the mean difference in metabolomic marker concentration in SD per 1-SD increase in TSH and fT4 levels with 95% CI. For MR analyses on CAD, results are presented as odds ratio (OR) per 1-SD genetically determined increase in circulating TSH and fT4 levels with 95% confidence interval (CI). As all MR analyses were hypothesis-driven, a conventional two-sided *p* value of less than 0.05 was considered statistically significant.

All analyses and data visualization were performed in R version 3.6.1 [45] supplemented with the following packages; MRCIEU/TwoSampleMR [46], rondolab/MR-PRESSO [44], metafor [47], ggplot2 [48], and ggforestplot [49].

## Results

### Associations between TSH and fT4 within the reference range and metabolomic profile

#### Participant characteristics of the stage 1 cohorts

For the multivariable regression analyses, 11,140 adults from six cohorts were included. A total of 9432 (84.7%) were euthyroid, 194 (1.7%) had hypothyroidism, 721 (6.5%) had subclinical hypothyroidism, 263 (2.4%) had subclinical hyperthyroidism, and 54 (0.5%) had hyperthyroidism (Additional File 1: Online Table 2). Among euthyroid individuals, the median age varied from 23.0 to 75.4 years and 54.5% of these participants were women (Table 1). Median TSH levels ranged between 1.73 and 2.13 mIU/L, mean fT4 levels ranged between 15.6 to 16.4 pmol/L, and thyroid medication was used by 185 individuals (2.0%) and lipid-modifying medication by 2694 individuals (28.6%).

**Table 1:** Population characteristics of biochemically euthyroid individuals in included cohorts (*n* = 9432)

	500 FG	GARP	LLS	NESDA	PROSPER	RS
	*N* = 362	*N* = 230	*N* = 419	*N* = 2467	*N* = 4513	*N* = 1441
Age in years (median (IQR))	23.0 (21.0–26.0)	59.8 (55.1–65.5)	65.7 (61.8–70.4)	43.0 (30.0–53.0)	75.4 (72.9–78.3)	68.9 (65.2–73.3)
Women	200 (55.2)	181 (78.7)	202 (48.2)	1594 (64.6)	2195 (48.6)	773 (53.6)
Current smoker	47 (13.1)^a^	38 (16.5)	51 (12.2)^c^	984 (39.9)	1231 (27.3)^g^	204 (14.2)^h^
BMI (median (IQR))	22.3 (20.8–24.2)^b^	26.0 (24.0–29.0)	26.3 (24.2–28.6)^d^	24.6 (22.0–28.0)^f^	26.2 (23.8–28.9)^g^	26.4 (24.2–29.0)
TSH (median (IQR))	2.09 (1.59–2.79)	1.76 (1.27–2.34)	2.13 (1.54–2.89)	2.07 (1.47–2.80)	1.73 (1.22–2.44)	1.76 (1.27–2.51)
fT4 (mean (SD))	16.4 (2.1)	15.8 (1.8)	15.6 (1.9)	15.6 (2.0)	15.6 (1.9)	15.7 (1.8)
History of diabetes mellitus	0 (0.0)	3 (1.3)	20 (6.0)^e^	103 (4.2)	471 (10.4)	149 (10.4)^i^
Lipid-lowering medication use	0 (0.0)	8 (3.5)	55 (16.6)^e^	184 (7.5)	2248 (49.8)	199 (14.7)^j^
History of thyroid disease	0 (0.0)	N.A.	N.A.	62 (2.5)	N.A.	111 (7.7)
Thyroid medication use	0 (0.0)	3 (1.3)	7 (2.1)^e^	34 (1.4)	113 (2.5)	28 (1.9)
Medication use influencing the thyroid gland	0 (0.0)	N.A.	1 (0.3)^e^	3 (0.1)	12 (0.3)	N.A.

Results are shown as *n* (%) unless indicated otherwise. *Abbreviations*: *500 FG* 500 Functional Genomics Study, *GARP* the Genetics, Arthrosis and Progression study, *LLS* the Leiden Longevity Study, *NESDA* the Netherlands Study of Depression and Anxiety, *PROSPER* PROspective Study of Pravastatin in the Elderly at Risk, *RS* the Rotterdam Study, *BMI* body mass index, *TSH* thyroid stimulating hormone, *fT4* free thyroxin, *N.A.* not available

^a^Information on 360 individuals

^b^Information on 356 individuals

^c^Information on 410 individuals

^d^Information on 407 individuals

^e^Information on 331 individuals

^f^Information on 2465 individuals

^g^Information on 4511 individuals

^h^Information on 1435 individuals

^i^Information on 1438 individuals

^j^Information on 1352 individuals

#### Stage 1 analyses

**Fig. 1:**
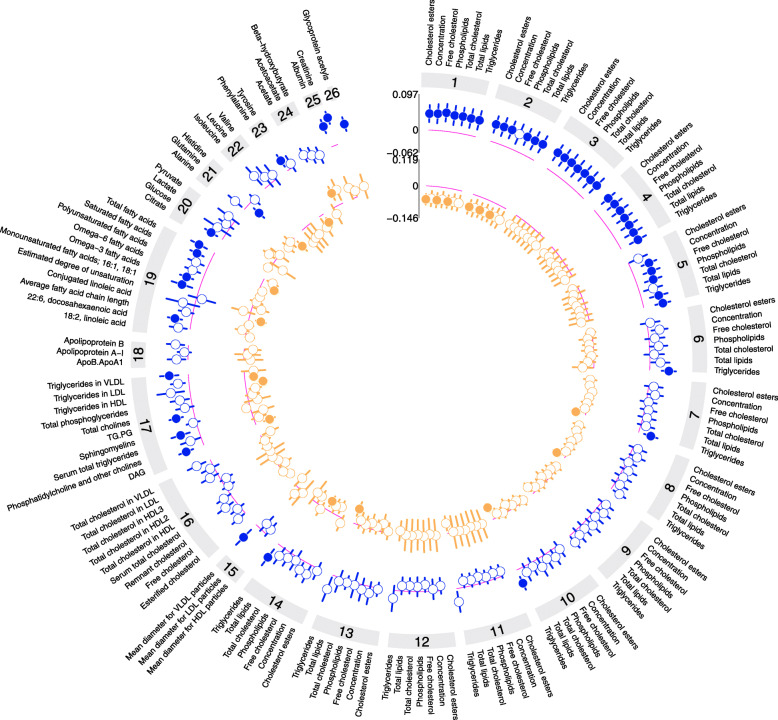
First stage associations between standardized TSH and fT4 within the reference range and 161 Nightingale platform metabolomic markers (*N* = 9353). Point estimates represent the standardized change in metabolomic marker concentration per standard deviation change in TSH, adjusted for age, sex, body mass index, and smoking. Red bars indicate positive associations; blue bars indicate negative associations. Hollow effect estimates were not statistically significant after correction for multiple testing (*p* value < 1.34 × 10^−3^). (1) Extreme large VLDL. (2) Very large VLDL. (3) Large VLDL. (4) Medium VLDL. (5) Small VLDL. (6) Very small VLDL. (7) IDL. (8) Large LDL. (9) Medium LDL. (10) Small LDL. (11) Very large HDL. (12) Large HDL. (13) Medium HDL. (14) Small HDL. (15) Lipoprotein particle size. (16) Cholesterol. (17) Glycerides and phospholipids. (18) Apolipoproteins. (19) Fatty acids. (20) Glycolysis-related metabolites. (21) Amino acids. (22) Branched-chain amino acids. (23) Aromatic amino acids. (24) Ketone bodies. (25) Fluid balance. (26) Inflammation. HDL, high-density lipoprotein; LDL, low-density lipoprotein; VLDL, very-low density lipoprotein

TSH levels were associated with 52/161 metabolomic marker concentrations and fT4 levels associated with 21/161 metabolomic markers (Fig. 1; Additional File 1: Online Table 3). Higher TSH levels were predominantly associated with higher concentrations of very low-density lipoprotein (VLDL) subclasses and components, higher triglyceride concentrations, and higher triglyceride content of lipoproteins. Associations with fT4 were largely an inverse reflection of those observed with TSH. Fluid balance parameters (creatinine and albumin) appeared specific for TSH, while ketone bodies appeared specific for fT4.

#### Stage 2 analyses

**Fig. 2:**
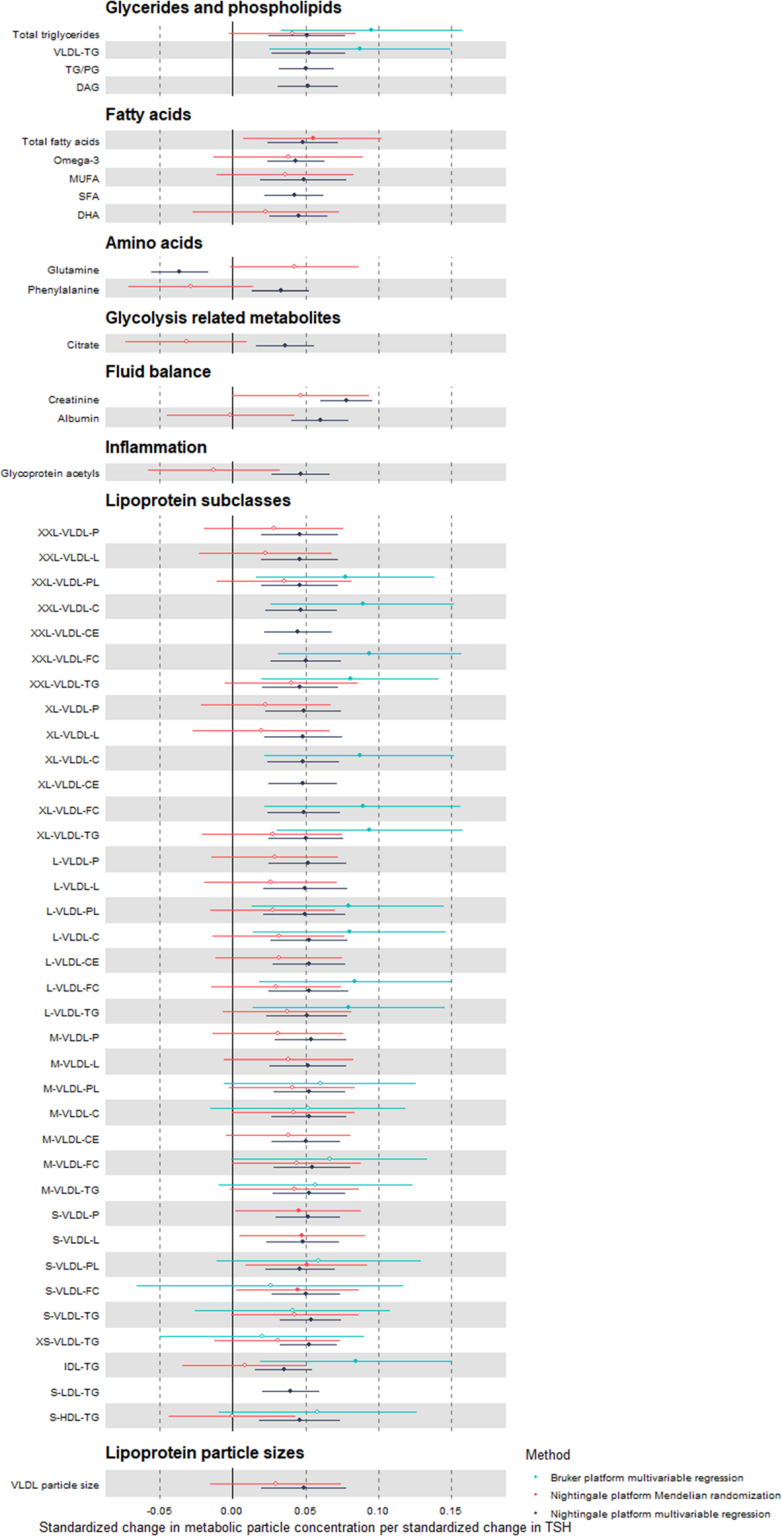
Second stage associations between TSH and 52 metabolomic markers using multivariable and Mendelian randomization analyses on Nightingale platform and multivariable analysis on Bruker platform. Point estimates represent the standardized change in metabolomic marker concentration per standard deviation change in TSH; error bars indicate 95% confidence intervals. Multivariable analyses were adjusted for age, sex, body mass index, and smoking, Mendelian randomization analyses are inverse variance-weighted (IVW) estimate. Hollow effect estimates refer to associations with *p* value > 0.05

For the metabolomic markers associated with TSH in the first stage, second-stage analyses with MR and/or Bruker platform were performed to assess directional consistency of the results (Fig. 2; Additional File 1: Online Table 4). For the MR meta-analysis of TSH and metabolomic markers, 41/52 metabolomic markers identified in the first stage were available. For the majority of these (34/41), associations observed with MR and multivariable regression were directionally consistent. These markers included various subclasses of VLDL cholesterol, fatty acids, and triglyceride subclasses. Inconsistent associations between MR and multivariable regression comprised associations of TSH with triglyceride content of IDL- and small HDL-cholesterol particles, albumin, various amino acids, glycolysis related metabolites, and inflammatory markers. Overlapping coverage between Nightingale and Bruker was found for 23/52 of the metabolomic markers identified in the first stage that all showed comparable associations in multivariable regression analyses across both platforms.

For the metabolomic markers associated with fT4 in the first stage, second-stage analyses were performed to assess robustness (Additional File 1: Online Figure 1; Additional File 1: Online Table 4). Of the only 4/21 metabolomic markers from the first stage present in available genetics data, the association with acetoacetate was directionally consistent in MR, but the observations with the amino acids and triglyceride content of IDL-cholesterol were not. A total of 9/21 markers were present on both the Nightingale and Bruker platforms (e.g., VLDL cholesterol subclass, HDL and LDL triglyceride content); all showed directional consistency with similar effect estimates.

Restricting the study sample to those without thyroid or lipid-lowering medication use or metabolic disease, produced similar results as observed in our main analyses (Additional File 1: Online Table 5). Sensitivity analyses for MR and the MR findings on the Bruker platform were consistent with the main findings (Additional File 1: Online Table 6).

### Association between biochemical thyroid dysfunction and metabolomic markers identified in relation to TSH and fT4

Consistency of the observed metabolomic profile was additionally explored in individuals with hyperthyroidism (*n* = 54) and hypothyroidism (*n* = 194). Virtually all metabolomic markers identified in the first stage analyses with TSH and fT4 were directionally consistent with hypo- and hyperthyroidism (Additional File 1: Online Figure 2). For TSH, 44/52 and 5/52 and for fT4, 14/21 and 2/31 metabolomic markers reached nominal significant associations (*p* < 0.05) with respectively hypothyroidism and hyperthyroidism (Additional File 1: Online Table 7). Overall, the VLDL subclasses and components associated with TSH and fT4 within the reference range appeared to associate stronger with hypothyroidism than with hyperthyroidism.

### Associations between genetically determined TSH and fT4 and coronary artery disease

**Fig. 3:**
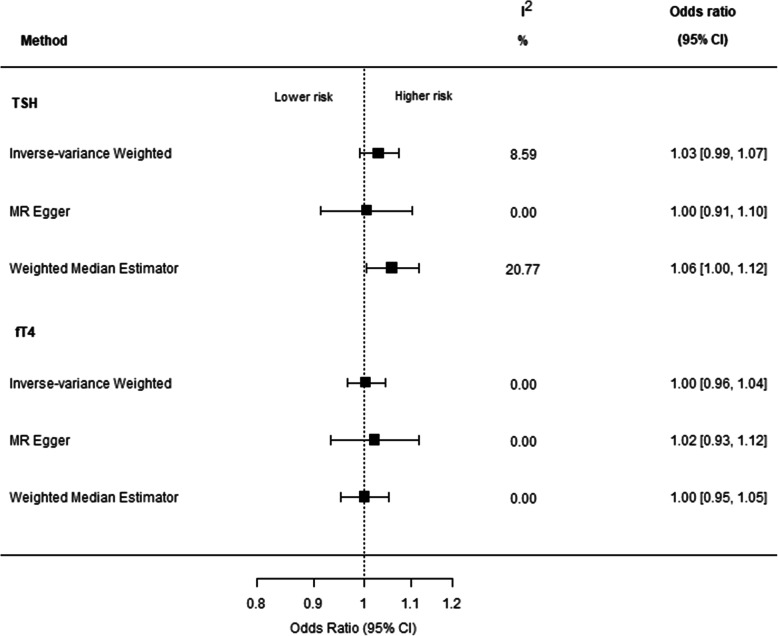
Associations between genetically determined standardized TSH and fT4 within the reference range and coronary artery disease (91,810 CAD cases and 656,091 controls of European ancestry). Odds ratios (ORs) shown (per 1 s.d. increase in TSH/fT4 concentration) are inverse variance-weighted (IVW) estimate, MR Egger, and weighted-median estimator; error bars indicate 95% confidence intervals

Within the multi-cohort MR study comprising 91,810 cases with CAD and 656,091 controls, per 1-SD increase of genetically determined TSH concentration CAD risk increased with an OR of 1.03 (95% CI 0.99–1.07; *p* value 0.16) (Fig. 3). Genetically determined fT4 concentrations were not associated with CAD (OR 1.00 per 1-SD increase of genetically determined fT4; 95% CI 0.96–1.04; *p* value 0.89). Heterogeneity between cohorts was low; all study-level effect estimates were congruent and *I*^2^ < 21.00%. The MR Egger and WME were consistent with the IVW estimates (Fig. 3), although some evidence was observed in the meta-analysis that higher TSH was associated with higher CAD risk (OR 1.06 per 1-SD increase of genetically determined TSH; 95% CI 1.00–1.10). The MR Egger intercepts did not deviate from zero and MR-PRESSO did not indicate distortion by outliers (Additional File 1: Online Table 8).

## Discussion

Using a mixed-methods approach of multi-cohort multivariable regression analysis and MR, we identified a robust metabolomic profile associated with lower thyroid status within the reference range, comprising higher concentrations of VLDL subclasses and components, higher triglyceride concentrations, and higher triglyceride content of lipoproteins. These associations were directionally consistent in patients with thyroid disorders. In addition, in this multi-cohort MR study on thyroid status and CAD in an exclusively European-ancestry population, genetically determined higher TSH concentrations were associated with a slightly higher CAD risk (particularly in the WME analysis).

As far as we are aware, this is the first multi-cohort study investigating the association between thyroid status and metabolomic profile. Although various smaller studies were performed previously, results were difficult to compare due to the variety of platforms, techniques, and sample types (i.e., plasma, serum, urine) [50]. Nevertheless, in line with our results, most studies indicated a role of thyroid hormones in lipid metabolism. No MR studies on thyroid status parameters and detailed metabolomic profile have been published to date.

Previous multi-ancestry MR studies found no evidence for an association between thyroid status and CAD [9–11]. While one MR study found evidence for an association between hypothyroidism and CAD, no associations were observed between genetically determined TSH or fT4 concentrations and CAD [12]. We hypothesize that the difference between our and previous studies might be because we restricted our analyses to European-ancestry individuals to decrease the risk of population stratification bias. Future research should assess the extent of confounding by population stratification in MR studies on thyroid status.

Multiple mechanisms may underlie the observed associations between thyroid status, CAD, and metabolomic profile. An important function of thyroid hormone is to stimulate the mobilization and breakdown of cholesterol and bile acids as well as the de-novo synthesis of fatty acids and their uptake by peripheral tissues, especially oxidative tissues such as skeletal muscle, heart, and liver [51]. Thyroid status could alter hepatic clearance of lipoproteins and reverse transport of cholesterol [52]. Consequently, disturbances in thyroid hormone availability and action may result in disturbances in the balance between lipid mobilization/synthesis on the one hand and uptake/clearance on the other hand (reviewed by Duntas et al. [53]). In case of higher TSH/lower thyroid hormone, the rate of cholesterol mobilization will be higher than the rate of its degradation, resulting in higher circulating cholesterol levels, which form a substrate for lipid peroxidation and may enhance oxidative stress as well as low grade chronic inflammation. In parallel, higher TSH/lower fT4 may also result in a decreased clearance of TG-rich lipoproteins, which may further aggravate the adverse lipid profile. Lower thyroid status could therefore result in accumulation of fatty acids in VLDL particles and free triglycerides in the circulation, resulting in the observed metabolomic profile. Interestingly, the metabolomic profile that we observed for lower thyroid status resembles that identified previously for myocardial infarction [54]. Therefore, a plausible pathway would be from low thyroid status via unfavorable lipid profile which could provide a substrate for oxidative stress and inflammatory processes to CAD. Although several other potential mediating factors should be considered, including endothelial dysfunction, hypertension and alterations in coagulation [55].

The stronger effect of TSH compared to fT4 on CAD risk in our study should be interpreted with caution. Though the genetic variants for fT4 were all strongly associated with higher circulating fT4 levels, some of these genes do not result in higher intracellular thyroid hormone signaling [56]. These shortcomings of the fT4 genetic risk score were also demonstrated previously in context of thyroid status and atrial fibrillation [57]. Furthermore, interpretation of the association for genetically determined higher TSH with CAD cannot be specified to either variation within the reference range or including (sub)clinical hypothyroidism, as many of the genetic variants associated with higher TSH within the reference range also associated with TSH levels above the reference range [16].

The present study has a number of strengths. Owing to the multi-cohort setting, we could compile large study populations for our analyses. Beside the statistical benefits of large sample sizes, multi-cohort studies allow for surpassing cohort-specific effects and therefore contribute to identifying robust and generalizable associations. Apart from assessing consistency of associations between study populations and the possibility of neglecting some important confounders not present in all contributing cohorts (e.g., specific drug use), we made efforts to triangulate our findings, using different epidemiological research methods, on the metabolomic profile. To assess (biological) consistency and robustness, the metabolomic markers associated with variation in TSH and fT4 within the reference range were tested in individuals with thyroid disorders and in studies using another NMR metabolomic profiling platform. The directional consistency among these different approaches indicates robust results.

Our study also has certain limitations. The MR study on the association of TSH and fT4 with CAD was performed in European-ancestry individuals only and is therefore not directly extrapolatable to other ethnicities. Furthermore, although we attempted to include as much cohorts as possible in our study, cohorts with both exposure and outcome were scarce and therefore the power of some of the analyses, in particular the validation analyses, is limited. Despite claims for causal inference in MR studies, caution is warranted for bias due to horizontal pleiotropy, selection bias and latent structure [58–60]. The study population used for first stage analyses of associations between TSH and fT4 and metabolomic profile included a considerable proportion of individuals using lipid-lowering medication (30%) or with a history of DM (8%). Nevertheless, results from our second stage and sensitivity analyses excluding participants with thyroid or lipid-lowering medication and those with a history of thyroid disease or DM were in line with our first stage results. However, not all metabolomic markers could be tested in the second stage analysis due to low overlap of markers in available data and platforms. Furthermore, both the MR study on genetically determined TSH and fT4 with CAD and with circulating metabolites suffered from some sample overlap between exposure and outcome study populations, which might cause bias, though the extent appears limited [61]. Moreover, multivariable MR to formally assess mediation of the association between TSH and CAD by metabolomic profile was not possible, as specific genetic instruments for separate metabolomic markers are currently unavailable due to the high (genetic) correlation between the different components and subclasses.

## Conclusions

We found indications for potentially causal elevated risks of unfavorable lipid profile and a somewhat increased risk cardiovascular disease in individuals with TSH on the upper limits of the reference range. However, the effect sizes were small and therefore do not justify widespread treatment with levothyroxine for prevention of cardiovascular disease. Nevertheless, the present study adds novel insights in the cardiovascular risk profile of those with altered thyroid hormone levels.

## Supplementary Information

**Additional file 1: **Extended Methods. **Supplementary Table 1.** Associations of individual genetic instruments for TSH and fT4 with CAD. **Supplementary Table 2.** Population characteristics of included cohorts (n=11,140. **Supplementary Table 3.** First stage associations between standardized TSH and fT4 within the reference range and 161 metabolomic markers. **Supplementary Table 4.** Second stage associations between metabolomic markers associated with TSH and fT4 in Mendelian randomization analyses and Bruker platform. **Supplementary Table 5.** Sensitivity analyses for metabolomic markers associated with TSH and fT4 in a restricted population without thyroid medication, lipid-lowering medication or history of diabetes. **Supplementary Table 6.** Sensitivity analyses for Mendelian randomization analyses of metabolomic markers and TSH and fT4. **Supplementary Table 7.** Associations between metabolomic markers associated with TSH and fT4 and biochemical thyroid dysfunction. **Supplementary Table 8.** Results for sensitivity analyses for MR on thyroid status and CAD. **Supplementary Figure 1.** Second stage associations between fT4 and 21 metabolomic markers. **Supplementary Figure 2.** Association of thyroid dysfunction with metabolomic markers identified for TSH and fT4 in first stage.

## Data Availability

The data used for the analyses on CAD are all publicly available. The summary-level data for CARDIoGRAM (http://www.cardiogramplusc4d.org/data-downloads/) and FinnGen (https://finngen.gitbook.io/documentation/data-download) are freely obtainable. The data for UK Biobank are accessible after approval (https://www.ukbiobank.ac.uk/). All other datasets used and/or analyzed during the current study are available from the corresponding authors on reasonable request.

## Abbreviations

500FG: 500 Functional Genomics Study
Airwave: Airwave Health Monitoring Study
ATC: Anatomical Therapeutic Chemical
BMI: Body mass index
CAD: Coronary artery disease
CARDIoGRAM: Coronary Artery Disease Genome wide Replication and Meta-analysis
CI: Confidence interval
DM: Diabetes mellitus
fT4: Free thyroxine
GARP: Genetics, Arthrosis and Progression study
GWAS: Genome-wide association study
HDL: High-density lipoprotein
IDL: Intermediate-density lipoprotein
IQR: Interquartile range
IVW: Inverse-variance weighted
LDL: Low-density lipoprotein
LLS: Leiden Longevity Study
MR: Mendelian randomization
MR-PRESSO: Mendelian randomization pleiotropy residual sum and outlier
NEO: Netherlands Epidemiology of Obesity
NESDA: Netherlands Study of Depression and Anxiety
NMR: Nuclear magnetic resonance
OR: Odds ratio
PROSPER: Prospective Study of Pravastatin in the Elderly at Risk
REML: Restricted maximum likelihood
RS: Rotterdam Study
SD: Standard deviation
SHIP: Study of Health in Pomerania
SNP: Single nucleotide polymorphism
TSH: Thyrotropin/thyroid-stimulating hormone
WME: Weighted median estimator
VLDL: Very low-density lipoprotein
