# Supplementary material for: Higher thyrotropin leads to unfavorable lipid profile and somewhat higher cardiovascular disease risk: evidence from multi-cohort Mendelian randomization and metabolomic profiling
Source: BMC Med. 2021 Nov 3;19:266. doi: 10.1186/s12916-021-02130-1 (PMC8565073; doi:10.1186/s12916-021-02130-1)
Supplement: Supplementary file 1 — Additional file 1: Extended Methods. Supplementary Table 1. Associations of individual genetic instruments for TSH and fT4 with CAD. Supplementary Table 2. Population characteristics of included cohorts (n=11,140. Supplementary Table 3. First stage associations between standardized TSH and fT4 within the reference range and 161 metabolomic markers. Supplementary Table 4. Second stage associations between metabolomic markers associated with TSH and fT4 in Mendelian randomization analyses and Bruker platform. Supplementary Table 5. Sensitivity analyses for metabolomic markers associated with TSH and fT4 in a restricted population without thyroid medication, lipid-lowering medication or history of diabetes. Supplementary Table 6. Sensitivity analyses for Mendelian randomization analyses of metabolomic markers and TSH and fT4. Supplementary Table 7. Associations between metabolomic markers associated with TSH and fT4 and biochemical thyroid dysfunction. Supplementary Table 8. Results for sensitivity analyses for MR on thyroid status and CAD. Supplementary Figure 1. Second stage associations between fT4 and 21 metabolomic markers. Supplementary Figure 2. Association of thyroid dysfunction with metabolomic markers identified for TSH and fT4 in first stage. [file 12916_2021_2130_MOESM1_ESM.docx]

**Supplementary material**

Contents

[**Extended Methods** 3](#_Toc78305420)

[Description of GWAS from which genetic instruments were derived 3](#_Toc78305421)

[Study populations for multivariable regression analyses 3](#_Toc78305422)

[The 500 Functional Genomics Study (500FG) 3](#_Toc78305423)

[The Genetics, Arthrosis and Progression study (GARP) 3](#_Toc78305424)

[The Leiden Longevity Study (LLS) 3](#_Toc78305425)

[The Netherlands Study of Depression and Anxiety (NESDA) 4](#_Toc78305426)

[PROspective Study of Pravastatin in the Elderly at Risk (PROSPER) study 4](#_Toc78305427)

[The Rotterdam Study 4](#_Toc78305428)

[The Study of Health in Pomerania (SHIP) 5](#_Toc78305429)

[Study populations for Mendelian Randomization analyses 5](#_Toc78305430)

[Coronary ARtery DIsease Genome wide Replication and Meta-analysis (CARDIoGRAM) consortium 5](#_Toc78305431)

[UK Biobank 6](#_Toc78305432)

[FinnGen 6](#_Toc78305433)

[MAGNETIC consortium 6](#_Toc78305434)

[Netherlands Epidemiology of Obesity (NEO) Study 6](#_Toc78305435)

[Oxford Biobank 7](#_Toc78305436)

[PROspective Study of Pravastatin in the Elderly at Risk (PROSPER) study 7](#_Toc78305437)

[Airwave Health Monitoring Study 7](#_Toc78305438)

[Metabolomic profile measurements 8](#_Toc78305439)

[Nightingale platform 8](#_Toc78305440)

[Bruker Platform 8](#_Toc78305441)

[**Extended results** 10](#_Toc78305442)

[**Online Table 1.** 10](#_Toc78305443)

[**Online Table 2.** 14](#_Toc78305444)

[**Online Table 3.** 15](#_Toc78305445)

[**Online Table 4.** 22](#_Toc78305446)

[**Online Table 5.** 25](#_Toc78305447)

[**Online Table 6.** 27](#_Toc78305448)

[**Online Table 7.** 30](#_Toc78305449)

[**Online Table 8.** 33](#_Toc78305450)

[**Online Figure 1.** 34](#_Toc78305451)

[**Online Figure 2.** 35](#_Toc78305452)

[**References** 36](#_Toc78305453)

# **Extended Methods**

## Description of GWAS from which genetic instruments were derived

Genetic instruments for TSH and fT4 concentrations were extracted from the largest genome-wide association studies (GWAS) meta-analysis to date on thyroid function comprising up to 72,167 participants [16]. This GWAS excluded participants with TSH levels outside the cohort-specific reference range, participants of non-European ancestry, and participants using thyroid medication (Anatomical Therapeutic Chemical (ATC) code H03) and/or history of thyroid surgery. Across the cohorts included in the GWAS, the mean TSH approximated 1.81 mIU/L (standard deviation (SD) 1.52 mIU/L) and the mean fT4 was approximately 14.9 pmol/L (SD 2.4 pmol/L). Both TSH and fT4 levels were inverse normal transformed, to allow for comparability across cohorts.

## Study populations for multivariable regression analyses

### The 500 Functional Genomics Study (500FG)

500FG is a cohort study that aims to identify the host and environmental factors that influence heterogeneity in human immune responses[22]. In this context, multiple datasets were collected including deep immunological phenotyping, physiological parameters (among which hormone concentrations), and multi-omics data (including lipidome and metabolome). 500FG is a cohort of 500 healthy volunteers with caucasian genetic background: any chronic disease (including diabetes, hypercholesterolemia, endocrine diseases) were exclusion criteria. Measurements of TSH and fT4 were performed using electrochemiluminescence immunoassay (ECLIA) on the Cobas 8000 (E801) (Roche, Almere, the Netherlands) in a single batch. The reference ranges at our laboratory are 0.27-4.2 mU/L for TSH and 10.0-23.0 pmol/L for fT4. For TSH, the total coefficient of variation (CV) ranged between 3.5% at 0.17 mU/L and 2.1% at 2.3 mU/L (detection range 0.005-100 mU/L). The total CV for fT4 ranged between 2.6% at 18.4 pmol/L and 2.2% at 49.7 pmol/L (detection range 0.5-100 pmol/L).

### The Genetics, Arthrosis and Progression study (GARP)

The GARP study has been described in detail previously[23]. It aimed at identifying determinants of osteoarthritis and the progression of this disease. The study is based on sibships of white Dutch ancestry with clinical- and radiographically-confirmed osteoarthritis at two or more joint sites of the hand, spine (cervical or lumbar), knee or hip. In the current analyses we included 321 subjects from whom we had Nightingale metabolomics data and thyroid levels available. For measurements, blood samples were collected and stored at −80 °C until analysis. Measurements of TSH and fT4 were performed on serum using the Modular E170 (Roche, Almere, the Netherlands) at the Department of Clinical Chemistry of the Leiden University Medical Center. The reference ranges are 0.4-4.0 mU/L for TSH and 13.0-25.0 pmol/L for fT4.

### The Leiden Longevity Study (LLS)

The Leiden Longevity Study was enrolled from 2002 to 2006 and consists of 1671 members of long-lived families (mean age 60 years) and their 744 partners (mean age 60 years) as population controls. Members of long-lived families are very similar to the general populations, although they have a lower prevalence of cardiometabolic disease [24]. For the current study we have used the data of the inclusion period from 2009 and 2010, in which we obtained Nightingale metabolomics data and thyroid

measures from 246 members of long-lived families and 240 controls. Blood samples were collected after an overnight fast and stored (−80 °C) until analysis. Measurements of TSH and fT4 were performed using electrochemiluminescence immunoassay (ECLIA) on the Cobas 8000 (E602) from Roche, Almere, the Netherlands. The reference ranges at our laboratory are 0.3-4.8 mU/L for TSH and 12.0-22.0 pmol/L for fT4. For TSH, the interassay coefficient of variation (CV) ranged between 2.32% at 0.176 mU/L and 2.11% at 21.430 mU/L (detection range 0.005-100 mU/L). The interassay CV for fT4 ranged between 5.11% at 13.8 pmol/L and 6.23% at 42.1 pmol/L (detection range 0.3-100 pmol/L). The measurements were performed in a single batch at the Department of Clinical Chemistry of the Leiden University Medical Center.

### The Netherlands Study of Depression and Anxiety (NESDA)

The Netherlands Study of Depression and Anxiety (NESDA) is an observational longitudinal cohort study on the long term course and consequences of depressive and anxiety disorders[25]. In total 2,981 participants aged 18 to 65 years were recruited between 2004 and 2007 through different settings: community, primary care and specialized mental health clinics in order to obtain a representative sample of persons with and without depressive and anxiety disorders. After an overnight fast, baseline EDTA plasma samples were collected and stored in aliquots at -80°C until further analysis. Blood samples were analyzed in 2 batches (April 2014 and December 2014) by Brainshake Ltd./Nightingale Health. Markers of thyroid function were assessed at the day of the baseline interview. Serum TSH was measured by immunoluminometric assay on a random-access analyze (Roche Diagnostics, Germany). Serum fT4 was measured with a fluorescence immunoenzymometric assay on a random-access assay system (Roche Diagnostics, Germany).

### PROspective Study of Pravastatin in the Elderly at Risk (PROSPER) study

All data come from the PROspective Study of Pravastatin in the Elderly at Risk (PROSPER). A detailed description of the study has been published elsewhere[26]. PROSPER was a prospective multicenter randomized placebo-controlled trial to assess whether treatment with pravastatin diminishes the risk of major vascular events in elderly. Between December 1997 and May 1999, we screened and enrolled subjects in Scotland (Glasgow), Ireland (Cork), and the Netherlands (Leiden). Men and women aged 70-82 years were recruited if they had pre-existing vascular disease or increased risk of such disease because of smoking, hypertension, or diabetes. A total number of 5,804 subjects were randomly assigned to pravastatin or placebo. A large number of prospective tests were performed including Biobank tests and cognitive function measurements. In stored (−80 °C) plasma drawn from participant at 6 months, we measured TSH and free T4 in all available samples stored at a single center (University of Glasgow), using the same electrochemiluminescence immunodetection method on a Roche Elecsys 2010 (Burgess Hill, UK). The limit of detection of TSH was below 0.005 mIU/liter. The limit of detection of free T4 was 0.3 pmol/liter, with reference ranges of 12–22 pmol/liter.

### The Rotterdam Study

From 1989, all inhabitants aged 55 and older from a well-defined suburb in the city of Rotterdam, the Netherlands were invited to participate in the Rotterdam Study[27]. The initial cohort comprised 7,983 participants (RS-I) and was extended in 2000 (RS-II: 3,011 participants) and 2006 (RS-III: 3,932 participants, aged 45 years and older). In total, the Rotterdam Study comprises 14,926 participants aged 45 years or over. The overall response rate across the three waves was 72%. Between 2002 and 2014, metabolomics data was measured by Brainshake Ltd./Nightingale Health from blood samples. For this study we analyzed the plasma samples from RS 1 visit 4 and RS 2 visit 2 (2002-2005). The thyroid function measurements were performed in RS 1 visit 3 and RS 2 visit 1 (1997-2001). TSH and fT4 measurements were performed at the same time in both cohorts in serum sample stored at −80° C (the electrochemiluminescence immunoassay for thyroxine and thyrotropin, Roche, Mannheim, Germany). The reference range was 0.4-4.0 mIU/L for TSH and 11-15 pmol/L for fT4.

### The Study of Health in Pomerania (SHIP)

The Study of Health in Pomerania (SHIP) is a population-based study conducted in West Pomerania, a rural region in north-east Germany and a detailed description of the sampling procedure and the study population can be found elsewhere[28]. In total, 4420 subjects chose to participate (50.1% response). All participants gave written informed consent before taking part in the study. The study was approved by the ethics committee of the University of Greifswald and conformed to the principles of the declaration of Helsinki. SHIP data are publicly available for scientific and quality control purposes by application at www.community-medicine.de. For a subsample of 1000 subjects without self-reported diabetes plasma and urine metabolomic profiling data based on NMR were available. Self-reported history of thyroid disease was recorded comprising hyperthyroidism, hypothyroidism, goiter, thyroid nodules or others. Fasting blood samples were taken from the cubital vein of participants in the supine position between 7.00 a.m. and 12.00 p.m. In the same time span spot urine samples were taken. All samples were either analyzed immediately or stored at −80°C in the Integrated Research Biobank (LiCONiC, Lichtenstein). Serum levels of TSH, FT3 and FT4 were measured using an immunoassay (Dimension VISTA, Siemens Healthcare Diagnostics, Eschborn, Germany) with a functional sensitivity of 0.005 mU/l for TSH, 0.77 pmol/l for FT3 and 1.3 pmol/l for FT4. Plasma samples were stored frozen at -80°C until analysed. After thawing, 250 μl of plasma were mixed with 250 μl of phosphate buffer [prepared with D2O and contained sodium 3-trimethylsilyl-(2,2,3,3-D4)-1-propionate (TSP) as reference, (pH 7.4)]. For total, HDL- and LDL cholesterol as well as total triglycerides comparison with laboratory measurements yielded excellent correlations (all above 0.91). However, six subjects revealed as outlier in PCA were excluded from the data set. Similar to the data above, all values were log2-transformed prior statistical analyses.

## Study populations for Mendelian Randomization analyses

Coronary ARtery DIsease Genome wide Replication and Meta-analysis (CARDIoGRAM) consortium

Summary-level data was made publicly available of a meta-analysis of 14 GWAS of coronary artery disease (CAD) comprising 22,233 cases and 64,762 controls, all of European ancestry. CAD was defined as an inclusive diagnosis of myocardial infarction, angina pectoris, coronary revascularization or coronary stenosis >50%[37].

### UK Biobank

The UK Biobank cohort is a prospective general population cohort with 502,628 participants between the age of 40 and 70 years recruited from the general population between 2006 and 2010[38], more details can be found on the website (<https://www.ukbiobank.ac.uk>). We restricted the analyses to participants of European ancestry, who were in the full released imputed genomics databases (UK10K + HRC). CAD diagnoses were retrieved via linkage with the NHS database, reported according to the International Classification of Diseases 10^th^ Revision (ICD-10) coding. CAD was defined as a composite outcome of angina pectoris (I20), myocardial infarction (I21 and I22), and acute and chronic ischemic heart disease (I24 and I25). In total, 52,946 cases and 393,549 controls were identified. We performed logistic regression analyses to assess the associations between genetic instruments and CAD, adjusted for age, sex and 10 principal components, and corrected for familial relationships using BOLT_LMM (v2.3.2).

### FinnGen

FinnGen study is an ongoing cohort study launched in 2017, bringing together Finnish universities, hospitals and hospital districts, national Institute for Health and Welfare, Blood Service, biobanks and international pharmaceutical companies and hundreds of thousands of Finns[39]. For the present study we used “major coronary heart disease (CHD)” as outcome, which comprised hospital data ICD-10 codes angina pectoris (I20), myocardial infarction (I21-I23), ischemic heart diseases (I24 and I25), cardiac arrest (I46) and other unattended or cause unknown sudden death (R96 and R98). More detailed information is provided online (<https://finngen.gitbook.io/documentation/>). The analyses were based on the FinnGen data freeze 5, which consists of 16,631 cases of major CHD and 197,780 controls with complete instruments-CHD associations.

### MAGNETIC consortium

We used publicly-available summary statistics from the MAGNETIC NMR GWAS dataset [29], which comprises the additive (per-allele) beta coefficients with accompanying standard errors of the associations between genome-wide SNPs and 123 metabolomic measures. In this GWAS meta-analysis data of ~20,000 individuals from 14 cohorts of European ancestry. The 123 metabolomic measures were quantified by an earlier version of the same high-throughput proton NMR metabolomics platform (Nightingale Health Ltd., Helsinki, Finland).

### Netherlands Epidemiology of Obesity (NEO) Study

The present study is a cross-sectional analysis of baseline measurements of the Netherlands Epidemiology of Obesity (NEO) study, a population-based cohort study. The NEO study started in 2008 and included 6,671 individuals aged 45–65 years, with an oversampling of individuals with overweight or obesity. The study design and population is described in more detail elsewhere[31]. Men and women living in the greater area of Leiden (in the West of the Netherlands) were invited to participate if they were aged between 45 and 65 years and had a self-reported BMI of 27 kg/m^2^ or higher. In addition, all inhabitants aged between 45 and 65 years from one municipality (Leiderdorp) were invited to participate irrespective of their BMI, allowing for a reference group with a normal BMI distribution. The Medical Ethical Committee of the Leiden University Medical Center (LUMC) approved the design of the study. All participants gave their written informed consent. Genotyping was performed in participants form European ancestry, using the Illumina HumanCoreExome-24 BeadChip (Illumina Inc., San Diego, California, United States of America). Subsequently, genotypes were imputed to the 1000 Genome Project reference panel (v3 2011).

### Oxford Biobank

The Oxford Biobank (OBB) is a population-based cohort study of randomly selected healthy men and women living in Oxfordshire, UK. The study includes 7185 individuals aged 30 to 50 years old. The exclusion criteria for the OBB were history of myocardial infarction, diabetes mellitus type 1 or 2, heart failure, untreated malignancy, other ongoing systemic diseases, or ongoing pregnancy. Study recruitment criteria and population characteristics are described in detail elsewhere[30]. The OBB protocol is approved by the Oxfordshire Clinical Research Ethics Committee and all participants have provided informed consent. Genotyping was performed using the Affymetrix Axium chip, which has been similarly used in the UK Biobank.

### PROspective Study of Pravastatin in the Elderly at Risk (PROSPER) study

Prospective multicenter randomized placebo-controlled trial to assess whether treatment with pravastatin diminishes the risk of major vascular events in elderly. Between December 1997 and May 1999, potential participants were screened and enrolled in Scotland (Glasgow), Ireland (Cork), and the Netherlands (Leiden). Men and women aged 70-82 years were recruited if they had pre-existing vascular disease or increased risk of such disease because of smoking, hypertension, or diabetes[26]. A total number of 5,804 subjects were randomly assigned to pravastatin or placebo. Participants were followed for an average 3.5 years. A whole genome wide screening has been performed. Of 5,763 subjects DNA was available for genotyping. Genotyping was performed with the Illumina 660K beadchip, after QC (call rate <95%) 5,244 subjects and 557,192 SNPs were left for analysis. These SNPs were imputed to 2.5 million SNPs based on the HAPMAP built 36 with MACH imputation software. Subsequent imputation of the genotyped data was executed with the HRC Michigan imputation. For the present study only the 2,343 participants from the placebo arm were included.

### Airwave Health Monitoring Study

The Airwave Health Monitoring Study was first established in 2004 as a large-scale cohort of police officers in Great Britain. A total of 53,114 participants were enrolled by end of baseline recruitment in March 2015. Initially, it was aimed to investigate health outcomes related to the use of Terrestrial Trunked Radio (TETRA). This cohort has been expanded to also investigate the health of workforces in more general. The rationale, design, and methods of this study can be found elsewhere[32]. Genotyping and metabolomics analysis have been carried out on part of sample collection. Genotyping was conducted on the Illumina Infinium HumanCoreExome-12v1-1 BeadChip. Samples with genotype call rate <97% and heterozygosity rate >3SD from the mean were removed during quality control process. Duplicated and second-degree relatives were also excluded. Markers with missing rate >2%, significant deviation from Hardy-Weinberg equilibrium (P<1E-5) or minor allele frequency less than 1% were removed. Genotype imputation was performed with reference to the 1000G reference panel. Metabolomic profiling was performed in 2,021 heparin plasma samples of the Airwave Health Monitoring Study.

## Metabolomic profile measurements

### Nightingale platform

For the main analyses we used a high-throughput proton NMR metabolomic profiling platform[29] (Nightingale Health Ltd., Helsinki, Finland) to quantify a maximum of 148 metabolomic particle concentrations in fasting serum samples. This method provides quantification of lipoprotein subclass profiling with lipid concentrations within 14 lipoprotein subclasses. The 14 subclass sizes were defined as follows: extremely large VLDL with particle diameters from 75 nm upwards and a possible contribution of chylomicrons, five VLDL subclasses (average particle diameters of 64.0 nm, 53.6 nm, 44.5 nm, 36.8 nm, and 31.3 nm), IDL (28.6 nm), three LDL subclasses (25.5 nm, 23.0 nm, and 18.7 nm), and four HDL subclasses (14.3 nm, 12.1 nm, 10.9 nm, and 8.7 nm). Within the lipoprotein subclasses, the following components were quantified: total cholesterol, total lipids, phospholipids, free cholesterol, cholesteryl esters, and triglycerides. The mean size for VLDL, LDL and HDL particles were calculated by weighting the corresponding subclass diameters with their particle concentrations. An additional 58 small molecules, including those involved in glycolysis, ketone bodies and several amino acids are also quantified. These belonged to classes of apolipoproteins, cholesterol, fatty acids, glycerides, phospholipids, amino acids, fluid balance, glycolysis-related metabolites, inflammation, and ketone bodies. Detailed experimentation and applications of the NMR metabolomics platform have been described previously[29], as well as representative coefficients of variations (CVs) for the metabolomic markers[29].

### Bruker Platform

For additional analyses on a different NMR platform, metabolomic profiles were generated by Bruker (Bruker Biospin, Rheinstetten, Germany). Spectra were recorded on a Bruker AVANCE-II 600 NMR spectrometer operated by TOPSPIN 3.2 software (both Bruker Biospin, Rheinstetten, Germany), equipped with 5-mm z-gradient probe (Bruker Biospin, Rheinstetten, Germany) and automated tuning and matching (ATMA) unit (Bruker Biospin, Rheinstetten, Germany). Specimens were automatically delivered to the spectrometer via SampleJet (Bruker Biospin, Rheinstetten, Germany) into standard 5 mm NMR tubes. The acquisition temperature was set to 310°K. A standard one-dimensional ^1^H-NMR pulse sequence with suppression of the water peak (NOESYPRESAT) was used. The sequence has the form –RD-gz,1-90°-t-90°-tm-gz,2-ACQ, where RD is the relaxation delay (4 s) t is a short delay (~3 µs), 90° represents the 90° RF hard pulse, tm is the mixing time (10 ms), gz,1 and gz,2 are the magnetic field z gradients both applied for 1 ms and ACQ is the acquisition period (2.7 s) collecting 98304 data points at a sweep width of 30ppm. The receiver gain is set at 90.5 for all experiments. For pre-processing, a line-broadening of 0.3 Hz, a zero-filling to produce 128k data points and a first-order phase correction of 0.0 was applied. Spectral processing included zero-filling, line-broadening, Fourier transform and referencing of the chemical shift and determination of the spectral intensity per 1mmol protons for quantitative referencing. Chemical shifts of plasma spectra were referenced to the CH_3_-group signal of alanine adjusting it to 1.48 ppm. Spectra were segmented into N = 450 consecutive integrated spectral regions (buckets) of fixed width covering the region from 0.3 ppm to 1.4 ppm. Two subregions, i.e. 1.31-1.35 ppm and 1.16-1.21 ppm where excluded from binning to avoid unwanted influences coming from lactate and ethanol CH_3_-group signals in later application of the method in typical population cohorts. Finally, the spectrum is submitted to data analysis for lipoprotein subclass analysis B.I.LISA^TM^ (Bruker BioSpin GmbH Germany).

# **Extended results**

**Online Table 1. Associations of individual genetic instruments for TSH and fT4 with CAD**

|  |  |  |  |  |  |  | **Exposure** | | |  |  | **CARDIoGRAM** | | **UK Biobank** | | **FinnGen** | |
| --- | --- | --- | --- | --- | --- | --- | --- | --- | --- | --- | --- | --- | --- | --- | --- | --- | --- |
| **SNP** | **Gene** | **Chr.** | **Position** | **A1** | **A2** | **AF1** | **beta** | **se** | **p-value** | **% explained** | **F-statistics** | **Log**  **Odds** | **se** | **Log**  **Odds** | **se** | **Log**  **Odds** | **se** |
| **TSH** |  |  |  |  |  |  |  |  |  |  |  |  |  |  |  |  |  |
| rs12089835 | *CAPZB* | 1 | 19771438 | T | C | 0.35 | 0.073 | 0.007 | 9.18E-26 | 0.242 | 109 | 0.029 | 0.015 | 0.010 | 0.007 | -0.018 | 0.014 |
| rs10917469 | *CAPZB* | 1 | 19843576 | A | G | 0.84 | 0.111 | 0.009 | 3.00E-35 | 0.331 | 152 | 0.039 | 0.019 | 0.013 | 0.009 | 0.005 | 0.020 |
| rs74804879 | *CAPZB* | 1 | 19862320 | T | C | 0.65 | 0.05 | 0.007 | 1.20E-14 | 0.114 | 51 | NA | NA | -0.003 | 0.007 | NA | NA |
| rs334725 | *NFIA* | 1 | 61610049 | A | G | 0.95 | 0.174 | 0.015 | 2.06E-31 | 0.288 | 135 | -0.001 | 0.031 | 0.014 | 0.015 | -0.055 | 0.046 |
| rs17020122 | *VAV3* | 1 | 108357391 | T | C | 0.09 | 0.104 | 0.011 | 1.62E-21 | 0.177 | 89 | -0.043 | 0.023 | -0.019 | 0.011 | 0.005 | 0.020 |
| rs13015993 | *IGFBP5* | 2 | 217625523 | A | G | 0.73 | 0.082 | 0.007 | 5.38E-32 | 0.265 | 137 | 0.011 | 0.015 | -0.002 | 0.007 | -0.023 | 0.013 |
| rs6724073 | *DIRC3* | 2 | 218236786 | T | C | 0.74 | 0.051 | 0.008 | 9.15E-11 | 0.100 | 41 | -0.026 | 0.019 | -0.013 | 0.007 | 0.011 | 0.013 |
| rs1663070 | *SYN2* | 3 | 12239852 | T | C | 0.74 | -0.046 | 0.007 | 2.49E-11 | 0.081 | 43 | 0.031 | 0.017 | -0.009 | 0.007 | -0.003 | 0.015 |
| rs28502438 | *TM4SF4* | 3 | 149220109 | T | C | 0.57 | 0.034 | 0.006 | 7.28E-09 | 0.057 | 32 | 0.026 | 0.016 | 0.011 | 0.006 | 0.006 | 0.014 |
| rs13100823 | *IGF2BP2* | 3 | 185514088 | T | C | 0.31 | -0.041 | 0.007 | 2.35E-09 | 0.072 | 34 | 0.017 | 0.015 | 0.030 | 0.007 | 0.012 | 0.014 |
| rs59381142 | *HES1* | 3 | 193916181 | A | G | 0.24 | -0.058 | 0.008 | 2.08E-13 | 0.123 | 53 | -0.007 | 0.020 | 0.008 | 0.007 | 0.004 | 0.017 |
| rs11732089 | *NR3C2* | 4 | 149665602 | T | C | 0.80 | 0.115 | 0.008 | 3.71E-47 | 0.423 | 207 | -0.005 | 0.017 | -0.003 | 0.008 | 0.025 | 0.020 |
| rs139424329 | *PDE8B* | 5 | 76495539 | A | G | 0.01 | -0.200 | 0.032 | 2.05E-10 | 0.079 | 39 | NA | NA | NA | NA | -0.002 | 0.049 |
| rs2127387 | *PDE8B* | 5 | 76532571 | A | G | 0.41 | 0.144 | 0.006 | 1.39E-127 | 1.003 | 576 | 0.006 | 0.014 | -0.011 | 0.007 | 0.024 | 0.013 |
| rs1265091 | *PSORS1C1* | 6 | 31108129 | T | C | 0.20 | 0.057 | 0.009 | 1.20E-10 | 0.104 | 40 | 0.030 | 0.021 | 0.008 | 0.008 | -0.002 | 0.020 |
| rs744103 | *VEGFA/*  *LOC100132354* | 6 | 43805362 | A | T | 0.69 | 0.092 | 0.007 | 9.35E-40 | 0.362 | 173 | 0.010 | 0.018 | 0.006 | 0.007 | 0.019 | 0.014 |
| rs9381266 | *VEGFA/*  *LOC100132354* | 6 | 43905037 | T | C | 0.74 | 0.073 | 0.007 | 9.18E-26 | 0.205 | 109 | 0.028 | 0.016 | 0.003 | 0.007 | 0.009 | 0.016 |
| rs9497965 | *SASH1* | 6 | 148521292 | T | C | 0.40 | 0.044 | 0.006 | 1.12E-13 | 0.093 | 54 | 0.014 | 0.015 | -0.002 | 0.006 | 0.007 | 0.014 |
| rs1079418 | *PDE10A* | 6 | 166047034 | A | G | 0.69 | 0.101 | 0.007 | 1.71E-47 | 0.436 | 208 | -0.013 | 0.017 | 0.006 | 0.007 | 0.011 | 0.015 |
| rs56009477 | *SLC25A37* | 8 | 23356964 | A | G | 0.84 | 0.052 | 0.008 | 4.02E-11 | 0.073 | 42 | NA | NA | -0.005 | 0.009 | 0.014 | 0.019 |
| rs2439301 | *NRG1* | 8 | 32433013 | A | G | 0.23 | -0.059 | 0.008 | 8.22E-14 | 0.123 | 54 | 0.001 | 0.016 | 0.006 | 0.008 | -0.010 | 0.015 |
| rs10957494 | *SULF1* | 8 | 70365025 | A | G | 0.69 | -0.040 | 0.007 | 5.51E-09 | 0.068 | 33 | NA | NA | -0.005 | 0.007 | -0.005 | 0.014 |
| rs118039499 | *TG* | 8 | 133771635 | A | C | 0.98 | 0.184 | 0.024 | 8.83E-15 | 0.133 | 59 | NA | NA | 0.044 | 0.021 | NA | NA |
| rs2739067 | *TG* | 8 | 133951991 | A | G | 0.60 | -0.042 | 0.006 | 1.28E-12 | 0.085 | 49 | -0.014 | 0.014 | 0.002 | 0.007 | -0.041 | 0.014 |
| rs10814915 | *GLIS3* | 9 | 4290544 | T | C | 0.44 | 0.042 | 0.006 | 1.28E-12 | 0.087 | 49 | 0.007 | 0.014 | -0.008 | 0.006 | 0.001 | 0.013 |
| rs9298749 | *C9orf92* | 9 | 16214340 | A | C | 0.59 | -0.039 | 0.006 | 4.02E-11 | 0.074 | 42 | -0.036 | 0.015 | 0.014 | 0.007 | -0.003 | 0.013 |
| rs494242 | *ABO* | 9 | 136145118 | C | T | 0.40 | 0.052 | 0.006 | 2.22E-18 | 0.130 | 75 | -0.069 | 0.015 | -0.024 | 0.007 | NA | NA |
| rs11255790 | *GATA3* | 10 | 8682180 | T | C | 0.30 | -0.041 | 0.007 | 2.35E-09 | 0.071 | 34 | 0.015 | 0.015 | -0.003 | 0.007 | -0.011 | 0.017 |
| rs4933466 | *PTEN* | 10 | 89849519 | A | G | 0.61 | 0.040 | 0.006 | 1.31E-11 | 0.076 | 44 | 0.009 | 0.020 | 0.007 | 0.007 | 0.009 | 0.013 |
| rs12284404 | *PRDM11* | 11 | 45228686 | A | G | 0.27 | -0.067 | 0.007 | 5.27E-22 | 0.177 | 92 | -0.036 | 0.015 | -0.002 | 0.007 | -0.002 | 0.014 |
| rs4445669 | *CADM1* | 11 | 115045237 | T | C | 0.46 | -0.040 | 0.006 | 1.31E-11 | 0.079 | 44 | 0.000 | 0.014 | -0.020 | 0.006 | -0.007 | 0.013 |
| rs7329958 | *SPATA13* | 13 | 24782080 | T | C | 0.35 | -0.044 | 0.007 | 1.63E-10 | 0.088 | 40 | -0.024 | 0.017 | -0.005 | 0.007 | 0.013 | 0.014 |
| rs398745 | *MBIP* | 14 | 36536181 | A | C | 0.59 | -0.052 | 0.006 | 2.22E-18 | 0.131 | 75 | -0.023 | 0.014 | 0.002 | 0.006 | 0.009 | 0.013 |
| rs11159482 | *TSHR* | 14 | 81490842 | T | C | 0.09 | 0.085 | 0.013 | 3.11E-11 | 0.118 | 43 | NA | NA | 0.001 | 0.014 | -0.050 | 0.027 |
| rs12893151 | *TSHR* | 14 | 81619945 | A | C | 0.22 | -0.062 | 0.008 | 4.59E-15 | 0.132 | 60 | NA | NA | 0.009 | 0.008 | -0.015 | 0.015 |
| rs8015085 | *ITPK1* | 14 | 93585331 | A | G | 0.21 | 0.067 | 0.008 | 2.76E-17 | 0.149 | 70 | NA | NA | 0.009 | 0.008 | 0.023 | 0.017 |
| rs17477923 | *FAM227B/FGF7* | 15 | 49711185 | T | C | 0.74 | 0.083 | 0.007 | 9.88E-33 | 0.265 | 141 | 0.018 | 0.016 | 0.007 | 0.007 | -0.016 | 0.014 |
| rs13329353 | *DET1* | 15 | 89113877 | T | C | 0.68 | 0.061 | 0.007 | 1.46E-18 | 0.162 | 76 | 0.007 | 0.016 | 0.002 | 0.007 | 0.018 | 0.014 |
| rs1045476 | *ADCY9* | 16 | 4015313 | A | G | 0.18 | 0.049 | 0.008 | 4.53E-10 | 0.071 | 38 | -0.004 | 0.023 | 0.000 | 0.008 | 0.012 | 0.017 |
| rs30227 | *MIR365A* | 16 | 14405428 | T | C | 0.61 | -0.047 | 0.006 | 2.38E-15 | 0.105 | 61 | -0.028 | 0.016 | -0.011 | 0.007 | -0.005 | 0.013 |
| rs17767491 | *MAF* | 16 | 79745487 | A | G | 0.68 | 0.088 | 0.007 | 1.52E-36 | 0.337 | 158 | -0.018 | 0.015 | 0.007 | 0.007 | -0.010 | 0.014 |
| rs1157994 | *BCAS3* | 17 | 59338574 | A | G | 0.05 | -0.090 | 0.016 | 9.28E-09 | 0.077 | 32 | NA | NA | 0.024 | 0.016 | 0.096 | 0.032 |
| rs1042673 | *SOX9* | 17 | 70121339 | A | G | 0.52 | -0.055 | 0.006 | 2.44E-20 | 0.151 | 84 | -0.019 | 0.014 | -0.004 | 0.006 | 0.019 | 0.013 |
| rs963384 | *SOX9* | 17 | 70369758 | T | C | 0.46 | 0.035 | 0.006 | 2.72E-09 | 0.061 | 34 | 0.013 | 0.015 | 0.000 | 0.006 | 0.006 | 0.013 |
| rs4804413 | *INSR* | 19 | 7222655 | T | C | 0.44 | 0.053 | 0.006 | 5.08E-19 | 0.138 | 78 | 0.000 | 0.014 | 0.016 | 0.006 | 0.008 | 0.013 |
| rs1203944 | *FOXA2* | 20 | 22596879 | T | C | 0.23 | -0.051 | 0.007 | 1.60E-13 | 0.092 | 53 | -0.031 | 0.017 | 0.002 | 0.008 | -0.033 | 0.016 |
| **fT4** |  |  |  |  |  |  |  |  |  |  |  |  |  |  |  |  |  |
| rs145019385 | *DIO1* | 1 | 54252139 | T | C | 0.98 | 0.181 | 0.032 | 7.74E-09 | 0.128 | 32 | NA | NA | -0.008 | 0.021 | 0.099 | 0.067 |
| rs2235544 | *DIO1* | 1 | 54375570 | A | C | 0.52 | 0.139 | 0.007 | 4.78E-88 | 0.965 | 394 | 0.017 | 0.016 | 0.009 | 0.006 | 0.009 | 0.013 |
| rs4954192 | *ACMSD* | 2 | 135632981 | T | C | 0.44 | -0.041 | 0.007 | 2.35E-09 | 0.083 | 34 | 0.002 | 0.015 | 0.001 | 0.007 | -0.027 | 0.013 |
| rs6785807 | *SOX2-OT* | 3 | 181718601 | A | G | 0.15 | -0.059 | 0.009 | 2.77E-11 | 0.089 | 43 | 0.012 | 0.023 | 0.000 | 0.009 | -0.010 | 0.015 |
| rs6854291 | *AADAT* | 4 | 170992760 | A | G | 0.10 | 0.117 | 0.011 | 1.01E-26 | 0.246 | 113 | NA | NA | -0.012 | 0.011 | -0.005 | 0.029 |
| rs10946313 | *ID4* | 6 | 19381386 | T | C | 0.63 | 0.046 | 0.007 | 2.49E-11 | 0.099 | 43 | -0.016 | 0.015 | -0.002 | 0.007 | -0.006 | 0.013 |
| rs9356988 | *SLC17A4* | 6 | 25777481 | A | G | 0.27 | -0.051 | 0.007 | 1.60E-13 | 0.103 | 53 | -0.003 | 0.016 | -0.012 | 0.007 | 0.001 | 0.014 |
| rs17185536 | *LOC728012* | 6 | 100620931 | T | C | 0.24 | 0.073 | 0.008 | 3.59E-20 | 0.194 | 83 | 0.011 | 0.017 | -0.007 | 0.007 | -0.017 | 0.016 |
| rs67583169 | *CA8* | 8 | 61212179 | C | G | 0.87 | 0.061 | 0.010 | 5.30E-10 | 0.084 | 37 | 0.021 | 0.021 | 0.003 | 0.009 | 0.001 | 0.016 |
| rs10119187 | *GLIS3* | 9 | 4223660 | T | C | 0.81 | 0.050 | 0.009 | 1.38E-08 | 0.077 | 31 | NA | NA | -0.003 | 0.008 | 0.002 | 0.018 |
| rs10739496 | *FOXE1* | 9 | 100552559 | T | C | 0.66 | 0.078 | 0.007 | 3.88E-29 | 0.273 | 124 | -0.015 | 0.015 | -0.001 | 0.007 | -0.007 | 0.014 |
| rs10818937 | *NEK6* | 9 | 127015440 | T | C | 0.31 | -0.048 | 0.007 | 3.51E-12 | 0.099 | 47 | -0.022 | 0.015 | 0.003 | 0.007 | -0.013 | 0.014 |
| rs4842131 | *LHX3* | 9 | 139092679 | T | C | 0.45 | -0.104 | 0.008 | 6.12E-39 | 0.535 | 169 | 0.039 | 0.022 | 0.008 | 0.006 | 0.010 | 0.013 |
| rs55679545 | *LHX3* | 9 | 139122363 | A | G | 0.27 | 0.044 | 0.008 | 1.90E-08 | 0.076 | 30 | -0.016 | 0.016 | 0.010 | 0.007 | -0.002 | 0.014 |
| rs11039355 | *FNBP4* | 11 | 47737501 | T | C | 0.34 | -0.039 | 0.007 | 1.26E-08 | 0.068 | 31 | 0.008 | 0.015 | -0.008 | 0.007 | -0.032 | 0.014 |
| rs4149056 | *SLCO1B1* | 12 | 21331549 | T | C | 0.84 | -0.051 | 0.009 | 7.28E-09 | 0.070 | 32 | 0.027 | 0.019 | 0.004 | 0.009 | 0.053 | 0.016 |
| rs225014 | *DIO2* | 14 | 80669580 | T | C | 0.64 | 0.054 | 0.007 | 6.08E-15 | 0.134 | 60 | 0.005 | 0.015 | 0.005 | 0.007 | 0.004 | 0.015 |
| rs12323871 | *DIO3OS* | 14 | 101852075 | T | C | 0.82 | -0.047 | 0.008 | 2.11E-09 | 0.065 | 35 | -0.005 | 0.022 | 0.006 | 0.008 | -0.012 | 0.016 |
| rs11626434 | *DIO3OS* | 14 | 101998443 | C | G | 0.36 | 0.058 | 0.007 | 5.87E-17 | 0.155 | 69 | -0.042 | 0.018 | -0.003 | 0.007 | NA | NA |
| rs12907106 | *USP3* | 15 | 63873658 | C | G | 0.27 | -0.041 | 0.007 | 2.35E-09 | 0.066 | 34 | NA | NA | 0.001 | 0.007 | -0.001 | 0.014 |
| rs8063103 | *SNX29* | 16 | 12703395 | C | G | 0.85 | -0.052 | 0.009 | 3.78E-09 | 0.069 | 33 | 0.040 | 0.023 | 0.012 | 0.009 | -0.028 | 0.021 |
| rs113107469 | *SLC25A52* | 18 | 29306737 | T | C | 0.03 | 0.200 | 0.022 | 4.91E-20 | 0.233 | 83 | NA | NA | 0.039 | 0.018 | -0.049 | 0.046 |
| rs56069042 | *MC4R* | 18 | 57914644 | A | G | 0.96 | 0.106 | 0.019 | 1.21E-08 | 0.086 | 31 | NA | NA | -0.001 | 0.017 | -0.025 | 0.032 |

Abbreviations: TSH; thyroid stimulating hormone, fT4; free thyroxine, SNP; single nucleotide polymorphism, Chr; chromosome number, A1; effect allele, A2; other allele, AF1; effect allele frequency, logOdds; log odds ratio, se: standard error, N.A.; not available

Estimates, standard errors and p-values were derived directly from summary data of the respective studies and data were harmonized to display estimates for the same allele across studies.

**Online Table 2. Population characteristics of included cohorts (n=11,140)**

|  | **500 FG** | **GARP** | **LLS** | **NESDA** | **PROSPER** | **RS** |
| --- | --- | --- | --- | --- | --- | --- |
|  | **N=421** | **N = 321** | **N = 486** | **N = 2,906** | **N = 5,316** | **N=1,690** |
| **Demographics** |  |  |  |  |  |  |
| Age in years (median(IQR)) | 23.0 (20.0-26.0) | 59.6 (54.3-65.4) | 65.9 (61.8 -70.5) | 43.0 (30.0 – 53.0) | 75.5 (72.9 – 78.4) | 69.0 (65.3-73.4) |
| Women | 240 (57.0) | 261 (81.3) | 245 (50.4) | 1,929 (66.4) | 2,729 (51.3) | 938 (55.5) |
| Current smoker | 52 (12.4)^a^ | 54 (16.8) | 60 (12.6)^d^ | 1,120 (38.5) | 1,400 (26.3)^g^ | 263 (15.6)^i^ |
| BMI (median(IQR)) | 22.2 (20.7-24.2)^b^ | 26.0 (24.0-30.0)^c^ | 26.3 (24.1 – 28.5)^e^ | 24.7 (22.1 – 28.1) | 26.2 (23.9 – 29.0)^h^ | 26.5 (24.2-29.1) |
| **Thyroid function** |  |  |  |  |  |  |
| TSH (median(IQR)) | 2.27 (1.67-3.20) | 1.97 (1.32-3.04) | 2.28 (1.57 – 3.28) | 2.18 (1.51 – 3.06) | 1.80 (1.19 – 2.66) | 1.83 (1.25-2.74) |
| fT4 (mean(SD)) | 16.4 (2.5) | 15.2 (2.2) | 15.5 (2.5) | 15.3 (2.5) | 15.5 (2.4) | 15.5 (2.3) |
| Biochemical euthyroidism | 362 (86.0) | 230 (71.7) | 419 (86.2) | 2,467 (84.9) | 4,513 (84.9) | 1,441 ((85.3) |
| Hypothyroidism | 55 (13.1) | 56 (17.4) | 41 (8.4) | 239 (8.2) | 392 (7.4) | 134 (7.9) |
| Subclinical | 54 (12.8) | 40 (12.5) | 29 (6.0) | 197 (67.8) | 301 (5.7) | 100 (5.9) |
| Overt | 1 (0.2) | 16 (5.0) | 12 (2.5) | 42 (1.4) | 89 (1.7) | 34 (2.0) |
| Hyperthyroidism | 2 (0.5) | 9 (2.8) | 6 (1.2) | 29 (1.0) | 214 (4.0) | 64 (3.8) |
| Subclinical | 2 (0.5) | 9 (2.8) | 2 (0.4) | 22 (0.8) | 169 (3.2) | 59 (3.5) |
| Overt | 0 (0.0) | 0 (0.0) | 4 (0.8) | 7 (0.2) | 38 (0.7) | 5 (0.3) |
| **Medical history** |  |  |  |  |  |  |
| History of diabetes | 0 (0.0) | 5 (1.6) | 20 (5.2)^f^ | 129 (4.4) | 569 (10.7) | 185 (11.0)^j^ |
| Lipid-lowering medication use | 0 (0.0) | 13 (4.0) | 60 (15.5)^f^ | 207 (7.1) | 2,630 (49.5) | 236 (14.9)^k^ |
| History of thyroid disease | 0 (0.0) | N.A. | N.A. | 102 (3.5) | N.A. | 151 (8.9) |
| Thyroid medication use | 0 (0.0) | 10 (3.1) | 15 (3.9)^f^ | 61 (2.1) | 231 (4.3) | 44 (2.6) |
| Medication use influencing the thyroid gland | 0 (0.0) | N.A. | 2 (0.5)^f^ | 7 (0.2) | 27 (0.5) | N.A. |

Results are shown as n (%) unless indicated otherwise. Abbreviations: 500 FG; 500 Functional Genomics Study, GARP; the Genetics, Arthrosis and Progression study, LLS; the Leiden Longevity Study, NESDA; the Netherlands Study of Depression and Anxiety, PROSPER; PROspective Study of Pravastatin in the Elderly at Risk, RS; the Rotterdam Study, BMI; body mass index, TSH; thyroid stimulating hormone, fT4; free thyroxine, N.A.; not available.
^a^ Information on 418 individuals, ^b^ Information on 412 individuals, ^c^ Information on 320 individuals, ^d^ Information on 477 individuals, ^e^ Information on 474 individuals, ^f^ Information on 386 individuals, ^g^ Information on 5,309 individuals, ^h^ Information on 5,314 individuals, ^i^ Information on 1681 individuals, ^j^ Information on 1687 individuals, ^k^ Information on 1589 individuals.

**Online Table 3. First stage associations between standardized TSH and fT4 within the reference range and 161 metabolomic markers**

|  |  | **TSH** | | | | **fT4** | | | |
| --- | --- | --- | --- | --- | --- | --- | --- | --- | --- |
| **Metabolic particle** | **Description** | **Pooled estimate** | **Pooled SE** | **P-value** | **I²** | **Pooled estimate** | **Pooled SE** | **P-value** | **I²** |
| XXL-VLDL-P | Concentration of chylomicrons and extremely large VLDL particles | 0.046 | 0.013 | 6.05E-04 | 11.8 | -0.056 | 0.018 | 1.57E-03 | 10.0 |
| XXL-VLDL-L | Total lipids in chylomicrons and extremely large VLDL | 0.046 | 0.013 | 6.04E-04 | 11.8 | -0.057 | 0.018 | 1.64E-03 | 9.9 |
| XXL-VLDL-PL | Phospholipids in chylomicrons and extremely large VLDL | 0.046 | 0.013 | 6.29E-04 | 11.7 | -0.055 | 0.018 | 2.54E-03 | 9.1 |
| XXL-VLDL-C | Total cholesterol in chylomicrons and extremely large VLDL | 0.047 | 0.013 | 1.86E-04 | 14.0 | -0.061 | 0.015 | 4.38E-05 | 16.7 |
| XXL-VLDL-CE | Cholesterol esters in chylomicrons and extremely large VLDL | 0.045 | 0.012 | 1.53E-04 | 14.3 | -0.061 | 0.012 | 9.06E-07 | 24.1 |
| XXL-VLDL-FC | Free cholesterol in chylomicrons and extremely large VLDL | 0.051 | 0.012 | 4.33E-05 | 16.7 | -0.061 | 0.017 | 2.34E-04 | 13.5 |
| XXL-VLDL-TG | Triglycerides in chylomicrons and extremely large VLDL | 0.046 | 0.013 | 5.13E-04 | 12.1 | -0.057 | 0.018 | 1.62E-03 | 9.9 |
| XL-VLDL-P | Concentration of very large VLDL particles | 0.048 | 0.013 | 2.91E-04 | 13.1 | -0.057 | 0.021 | 5.49E-03 | 7.7 |
| XL-VLDL-L | Total lipids in very large VLDL | 0.048 | 0.014 | 4.18E-04 | 12.5 | -0.056 | 0.021 | 9.46E-03 | 6.7 |
| XL-VLDL-PL | Phospholipids in very large VLDL | 0.046 | 0.015 | 1.75E-03 | 9.8 | -0.057 | 0.019 | 3.15E-03 | 8.7 |
| XL-VLDL-C | Total cholesterol in very large VLDL | 0.048 | 0.012 | 1.01E-04 | 15.1 | -0.058 | 0.017 | 7.40E-04 | 11.4 |
| XL-VLDL-CE | Cholesterol esters in very large VLDL | 0.048 | 0.012 | 6.32E-05 | 16.0 | -0.060 | 0.017 | 3.94E-04 | 12.6 |
| XL-VLDL-FC | Free cholesterol in very large VLDL | 0.049 | 0.013 | 1.14E-04 | 14.9 | -0.057 | 0.018 | 1.17E-03 | 10.5 |
| XL-VLDL-TG | Triglycerides in very large VLDL | 0.050 | 0.013 | 1.29E-04 | 14.7 | -0.058 | 0.021 | 6.69E-03 | 7.4 |
| L-VLDL-P | Concentration of large VLDL particles | 0.051 | 0.014 | 1.62E-04 | 14.2 | -0.050 | 0.024 | 3.97E-02 | 4.2 |
| L-VLDL-L | Total lipids in large VLDL | 0.050 | 0.015 | 6.65E-04 | 11.6 | -0.046 | 0.026 | 7.91E-02 | 3.1 |
| L-VLDL-PL | Phospholipids in large VLDL | 0.049 | 0.014 | 5.68E-04 | 11.9 | -0.048 | 0.024 | 4.75E-02 | 3.9 |
| L-VLDL-C | Total cholesterol in large VLDL | 0.052 | 0.014 | 1.10E-04 | 15.0 | -0.051 | 0.023 | 2.97E-02 | 4.7 |
| L-VLDL-CE | Cholesterol esters in large VLDL | 0.052 | 0.013 | 3.59E-05 | 17.1 | -0.051 | 0.022 | 2.22E-02 | 5.2 |
| L-VLDL-FC | Free cholesterol in large VLDL | 0.052 | 0.014 | 2.10E-04 | 13.7 | -0.052 | 0.023 | 2.22E-02 | 5.2 |
| L-VLDL-TG | Triglycerides in large VLDL | 0.051 | 0.014 | 3.50E-04 | 12.8 | -0.049 | 0.026 | 5.65E-02 | 3.6 |
| M-VLDL-P | Concentration of medium VLDL particles | 0.053 | 0.013 | 2.02E-05 | 18.2 | -0.039 | 0.027 | 1.40E-01 | 2.2 |
| M-VLDL-L | Total lipids in medium VLDL | 0.052 | 0.013 | 1.30E-04 | 14.6 | -0.036 | 0.028 | 2.00E-01 | 1.7 |
| M-VLDL-PL | Phospholipids in medium VLDL | 0.052 | 0.013 | 3.05E-05 | 17.4 | -0.038 | 0.027 | 1.66E-01 | 1.9 |
|  |  | **TSH** | | | | **fT4** | | | |
| **Metabolic particle** | **Description** | **Pooled estimate** | **Pooled SE** | **P-value** | **I²** | **Pooled estimate** | **Pooled SE** | **P-value** | **I²** |
| M-VLDL-C | Total cholesterol in medium VLDL | 0.052 | 0.013 | 5.48E-05 | 16.3 | -0.042 | 0.024 | 8.47E-02 | 3.0 |
| M-VLDL-CE | Cholesterol esters in medium VLDL | 0.050 | 0.012 | 2.82E-05 | 17.5 | -0.045 | 0.020 | 2.66E-02 | 4.9 |
| M-VLDL-FC | Free cholesterol in medium VLDL | 0.054 | 0.013 | 5.18E-05 | 16.4 | -0.048 | 0.025 | 4.88E-02 | 3.9 |
| M-VLDL-TG | Triglycerides in medium VLDL | 0.052 | 0.013 | 4.30E-05 | 16.7 | -0.037 | 0.028 | 1.77E-01 | 1.8 |
| S-VLDL-P | Concentration of small VLDL particles | 0.052 | 0.011 | 3.83E-06 | 21.4 | -0.032 | 0.028 | 2.50E-01 | 1.3 |
| S-VLDL-L | Total lipids in small VLDL | 0.048 | 0.013 | 1.72E-04 | 14.1 | -0.035 | 0.027 | 1.96E-01 | 1.7 |
| S-VLDL-PL | Phospholipids in small VLDL | 0.046 | 0.012 | 1.69E-04 | 14.1 | -0.038 | 0.027 | 1.57E-01 | 2.0 |
| S-VLDL-C | Total cholesterol in small VLDL | 0.038 | 0.013 | 3.56E-03 | 8.5 | -0.032 | 0.019 | 9.16E-02 | 2.9 |
| S-VLDL-CE | Cholesterol esters in small VLDL | 0.030 | 0.013 | 2.55E-02 | 5.0 | -0.026 | 0.016 | 1.16E-01 | 2.5 |
| S-VLDL-FC | Free cholesterol in small VLDL | 0.050 | 0.012 | 3.12E-05 | 17.3 | -0.046 | 0.023 | 4.16E-02 | 4.2 |
| S-VLDL-TG | Triglycerides in small VLDL | 0.053 | 0.011 | 6.22E-07 | 24.8 | -0.038 | 0.028 | 1.74E-01 | 1.9 |
| XS-VLDL-P | Concentration of very small VLDL particles | 0.032 | 0.011 | 5.14E-03 | 7.8 | -0.022 | 0.020 | 2.53E-01 | 1.3 |
| XS-VLDL-L | Total lipids in very small VLDL | 0.029 | 0.013 | 2.51E-02 | 5.0 | -0.028 | 0.017 | 1.05E-01 | 2.6 |
| XS-VLDL-PL | Phospholipids in very small VLDL | 0.025 | 0.012 | 3.94E-02 | 4.2 | -0.021 | 0.017 | 2.23E-01 | 1.5 |
| XS-VLDL-C | Total cholesterol in very small VLDL | 0.012 | 0.014 | 3.90E-01 | 0.7 | -0.011 | 0.014 | 4.13E-01 | 0.7 |
| XS-VLDL-CE | Cholesterol esters in very small VLDL | 0.009 | 0.013 | 4.96E-01 | 0.5 | -0.005 | 0.011 | 6.52E-01 | 0.2 |
| XS-VLDL-FC | Free cholesterol in very small VLDL | 0.016 | 0.013 | 2.26E-01 | 1.5 | -0.018 | 0.015 | 2.22E-01 | 1.5 |
| XS-VLDL-TG | Triglycerides in very small VLDL | 0.052 | 0.010 | 1.43E-07 | 27.7 | -0.051 | 0.023 | 2.65E-02 | 4.9 |
| IDL-P | Concentration of IDL particles | 0.021 | 0.011 | 5.47E-02 | 3.7 | -0.012 | 0.010 | 2.49E-01 | 1.3 |
| IDL-L | Total lipids in IDL | 0.014 | 0.012 | 2.29E-01 | 1.4 | -0.011 | 0.011 | 3.13E-01 | 1.0 |
| IDL-PL | Phospholipids in IDL | 0.011 | 0.011 | 3.50E-01 | 0.9 | -0.004 | 0.010 | 6.82E-01 | 0.2 |
| IDL-C | Total cholesterol in IDL | 0.011 | 0.012 | 3.57E-01 | 0.9 | -0.008 | 0.013 | 5.61E-01 | 0.3 |
| IDL-CE | Cholesterol esters in IDL | 0.014 | 0.012 | 2.53E-01 | 1.3 | -0.012 | 0.015 | 3.98E-01 | 0.7 |
| IDL-FC | Free cholesterol in IDL | 0.006 | 0.011 | 5.78E-01 | 0.3 | 0.005 | 0.010 | 6.46E-01 | 0.2 |
| IDL-TG | Triglycerides in IDL | 0.035 | 0.010 | 4.36E-04 | 12.4 | -0.046 | 0.010 | 5.01E-06 | 20.8 |
| L-LDL-P | Concentration of large LDL particles | 0.021 | 0.012 | 6.99E-02 | 3.3 | -0.012 | 0.012 | 2.82E-01 | 1.2 |
| L-LDL-L | Total lipids in large LDL | 0.014 | 0.013 | 2.72E-01 | 1.2 | -0.010 | 0.011 | 3.61E-01 | 0.8 |
|  |  | **TSH** | | | | **fT4** | | | |
| **Metabolic particle** | **Description** | **Pooled estimate** | **Pooled SE** | **P-value** | **I²** | **Pooled estimate** | **Pooled SE** | **P-value** | **I²** |
| L-LDL-PL | Phospholipids in large LDL | 0.016 | 0.013 | 2.04E-01 | 1.6 | -0.012 | 0.013 | 3.54E-01 | 0.9 |
| L-LDL-C | Total cholesterol in large LDL | 0.012 | 0.013 | 3.57E-01 | 0.9 | -0.008 | 0.014 | 5.66E-01 | 0.3 |
| L-LDL-CE | Cholesterol esters in large LDL | 0.014 | 0.013 | 2.75E-01 | 1.2 | -0.011 | 0.014 | 4.48E-01 | 0.6 |
| L-LDL-FC | Free cholesterol in large LDL | 0.005 | 0.012 | 6.68E-01 | 0.2 | 0.005 | 0.010 | 6.00E-01 | 0.3 |
| L-LDL-TG | Triglycerides in large LDL | 0.027 | 0.010 | 6.78E-03 | 7.3 | -0.043 | 0.010 | 1.62E-05 | 18.6 |
| M-LDL-P | Concentration of medium LDL particles | 0.023 | 0.012 | 4.72E-02 | 3.9 | -0.020 | 0.015 | 1.90E-01 | 1.7 |
| M-LDL-L | Total lipids in medium LDL | 0.016 | 0.013 | 2.06E-01 | 1.6 | -0.016 | 0.014 | 2.56E-01 | 1.3 |
| M-LDL-PL | Phospholipids in medium LDL | 0.023 | 0.014 | 8.47E-02 | 3.0 | -0.028 | 0.017 | 9.58E-02 | 2.8 |
| M-LDL-C | Total cholesterol in medium LDL | 0.013 | 0.013 | 3.16E-01 | 1.0 | -0.010 | 0.015 | 4.84E-01 | 0.5 |
| M-LDL-CE | Cholesterol esters in medium LDL | 0.014 | 0.013 | 2.88E-01 | 1.1 | -0.009 | 0.014 | 5.45E-01 | 0.4 |
| M-LDL-FC | Free cholesterol in medium LDL | 0.012 | 0.014 | 4.13E-01 | 0.7 | -0.016 | 0.016 | 3.24E-01 | 1.0 |
| M-LDL-TG | Triglycerides in medium LDL | 0.023 | 0.010 | 1.82E-02 | 5.6 | -0.037 | 0.012 | 1.96E-03 | 9.6 |
| S-LDL-P | Concentration of small LDL particles | 0.022 | 0.012 | 5.89E-02 | 3.6 | -0.022 | 0.014 | 1.26E-01 | 2.3 |
| S-LDL-L | Total lipids in small LDL | 0.017 | 0.013 | 1.86E-01 | 1.8 | -0.017 | 0.013 | 2.01E-01 | 1.6 |
| S-LDL-PL | Phospholipids in small LDL | 0.025 | 0.014 | 7.69E-02 | 3.1 | -0.032 | 0.015 | 2.99E-02 | 4.7 |
| S-LDL-C | Total cholesterol in small LDL | 0.011 | 0.013 | 3.91E-01 | 0.7 | -0.007 | 0.014 | 6.23E-01 | 0.2 |
| S-LDL-CE | Cholesterol esters in small LDL | 0.011 | 0.012 | 3.67E-01 | 0.8 | -0.004 | 0.013 | 7.39E-01 | 0.1 |
| S-LDL-FC | Free cholesterol in small LDL | 0.013 | 0.015 | 3.88E-01 | 0.8 | -0.015 | 0.015 | 3.33E-01 | 0.9 |
| S-LDL-TG | Triglycerides in small LDL | 0.040 | 0.010 | 5.38E-05 | 16.3 | -0.059 | 0.010 | 4.76E-09 | 34.3 |
| XL-HDL-P | Concentration of very large HDL particles | -0.017 | 0.009 | 8.08E-02 | 3.1 | -0.002 | 0.038 | 9.68E-01 | 0.0 |
| XL-HDL-L | Total lipids in very large HDL | -0.018 | 0.009 | 6.15E-02 | 3.5 | -0.002 | 0.038 | 9.57E-01 | 0.0 |
| XL-HDL-PL | Phospholipids in very large HDL | -0.019 | 0.011 | 7.50E-02 | 3.2 | 0.002 | 0.039 | 9.65E-01 | 0.0 |
| XL-HDL-C | Total cholesterol in very large HDL | -0.015 | 0.010 | 1.11E-01 | 2.5 | -0.001 | 0.035 | 9.80E-01 | 0.0 |
| XL-HDL-CE | Cholesterol esters in very large HDL | -0.014 | 0.010 | 1.42E-01 | 2.2 | 0.000 | 0.034 | 9.91E-01 | 0.0 |
| XL-HDL-FC | Free cholesterol in very large HDL | -0.012 | 0.010 | 1.91E-01 | 1.7 | -0.005 | 0.037 | 8.90E-01 | 0.0 |
| XL-HDL-TG | Triglycerides in very large HDL | 0.036 | 0.014 | 1.02E-02 | 6.6 | -0.047 | 0.017 | 5.50E-03 | 7.7 |
| L-HDL-P | Concentration of large HDL particles | -0.005 | 0.014 | 6.96E-01 | 0.2 | -0.005 | 0.037 | 8.89E-01 | 0.0 |
|  |  | **TSH** | | | | **fT4** | | | |
| **Metabolic particle** | **Description** | **Pooled estimate** | **Pooled SE** | **P-value** | **I²** | **Pooled estimate** | **Pooled SE** | **P-value** | **I²** |
| L-HDL-L | Total lipids in large HDL | -0.014 | 0.012 | 2.32E-01 | 1.4 | -0.001 | 0.036 | 9.68E-01 | 0.0 |
| L-HDL-PL | Phospholipids in large HDL | -0.014 | 0.013 | 3.08E-01 | 1.0 | -0.004 | 0.035 | 9.03E-01 | 0.0 |
| L-HDL-C | Total cholesterol in large HDL | -0.016 | 0.011 | 1.43E-01 | 2.1 | 0.000 | 0.038 | 9.93E-01 | 0.0 |
| L-HDL-CE | Cholesterol esters in large HDL | -0.013 | 0.011 | 2.30E-01 | 1.4 | 0.002 | 0.037 | 9.47E-01 | 0.0 |
| L-HDL-FC | Free cholesterol in large HDL | -0.019 | 0.010 | 6.03E-02 | 3.5 | 0.003 | 0.037 | 9.31E-01 | 0.0 |
| L-HDL-TG | Triglycerides in large HDL | 0.022 | 0.018 | 2.12E-01 | 1.6 | -0.018 | 0.023 | 4.25E-01 | 0.6 |
| M-HDL-P | Concentration of medium HDL particles | 0.019 | 0.016 | 2.40E-01 | 1.4 | -0.037 | 0.022 | 9.13E-02 | 2.9 |
| M-HDL-L | Total lipids in medium HDL | 0.010 | 0.016 | 5.23E-01 | 0.4 | -0.024 | 0.019 | 2.12E-01 | 1.6 |
| M-HDL-PL | Phospholipids in medium HDL | 0.007 | 0.018 | 6.96E-01 | 0.2 | -0.028 | 0.021 | 1.79E-01 | 1.8 |
| M-HDL-C | Total cholesterol in medium HDL | 0.006 | 0.010 | 5.14E-01 | 0.4 | -0.013 | 0.020 | 5.37E-01 | 0.4 |
| M-HDL-CE | Cholesterol esters in medium HDL | 0.004 | 0.010 | 7.09E-01 | 0.1 | -0.008 | 0.019 | 6.82E-01 | 0.2 |
| M-HDL-FC | Free cholesterol in medium HDL | 0.014 | 0.016 | 3.96E-01 | 0.7 | -0.017 | 0.016 | 3.04E-01 | 1.1 |
| M-HDL-TG | Triglycerides in medium HDL | 0.042 | 0.018 | 1.86E-02 | 5.5 | -0.070 | 0.017 | 5.31E-05 | 16.3 |
| S-HDL-P | Concentration of small HDL particles | 0.028 | 0.013 | 2.52E-02 | 5.0 | -0.044 | 0.016 | 5.09E-03 | 7.9 |
| S-HDL-L | Total lipids in small HDL | 0.024 | 0.013 | 6.72E-02 | 3.4 | -0.037 | 0.016 | 1.97E-02 | 5.4 |
| S-HDL-PL | Phospholipids in small HDL | 0.010 | 0.015 | 5.09E-01 | 0.4 | -0.045 | 0.014 | 8.92E-04 | 11.0 |
| S-HDL-C | Total cholesterol in small HDL | 0.016 | 0.013 | 2.28E-01 | 1.5 | 0.014 | 0.017 | 4.01E-01 | 0.7 |
| S-HDL-CE | Cholesterol esters in small HDL | 0.007 | 0.013 | 5.68E-01 | 0.3 | 0.025 | 0.019 | 1.98E-01 | 1.7 |
| S-HDL-FC | Free cholesterol in small HDL | 0.027 | 0.011 | 1.48E-02 | 5.9 | -0.036 | 0.017 | 4.22E-02 | 4.1 |
| S-HDL-TG | Triglycerides in small HDL | 0.046 | 0.014 | 1.05E-03 | 10.7 | -0.040 | 0.032 | 2.12E-01 | 1.6 |
| VLDL-D | Mean diameter for VLDL particles | 0.049 | 0.015 | 1.04E-03 | 10.8 | -0.043 | 0.025 | 8.53E-02 | 3.0 |
| LDL-D | Mean diameter for LDL particles | -0.022 | 0.014 | 1.12E-01 | 2.5 | 0.032 | 0.017 | 6.67E-02 | 3.4 |
| HDL-D | Mean diameter for HDL particles | -0.017 | 0.012 | 1.46E-01 | 2.1 | 0.004 | 0.037 | 9.04E-01 | 0.0 |
| Serum-C | Serum total cholesterol | 0.022 | 0.011 | 5.41E-02 | 3.7 | -0.013 | 0.010 | 1.85E-01 | 1.8 |
| VLDL-C | Total cholesterol in VLDL | 0.041 | 0.014 | 2.69E-03 | 9.0 | -0.049 | 0.020 | 1.31E-02 | 6.2 |
| Remnant-C | Remnant cholesterol (non-HDL. non-LDL -cholesterol) | 0.033 | 0.013 | 1.15E-02 | 6.4 | -0.032 | 0.016 | 4.78E-02 | 3.9 |
| LDL-C | Total cholesterol in LDL | 0.011 | 0.013 | 4.11E-01 | 0.7 | -0.007 | 0.014 | 5.88E-01 | 0.3 |
|  |  | **TSH** | | | | **fT4** | | | |
| **Metabolic particle** | **Description** | **Pooled estimate** | **Pooled SE** | **P-value** | **I²** | **Pooled estimate** | **Pooled SE** | **P-value** | **I²** |
| HDL-C | Total cholesterol in HDL | -0.009 | 0.009 | 3.47E-01 | 0.9 | 0.000 | 0.033 | 9.91E-01 | 0.0 |
| HDL2-C | Total cholesterol in HDL2 | -0.015 | 0.009 | 1.05E-01 | 2.6 | -0.005 | 0.036 | 8.86E-01 | 0.0 |
| HDL3-C | Total cholesterol in HDL3 | 0.024 | 0.013 | 6.48E-02 | 3.4 | -0.005 | 0.013 | 7.22E-01 | 0.1 |
| EstC | Esterified cholesterol | 0.025 | 0.011 | 1.85E-02 | 5.6 | -0.018 | 0.010 | 7.05E-02 | 3.3 |
| FreeC | Free cholesterol | 0.009 | 0.011 | 4.20E-01 | 0.7 | 0.001 | 0.010 | 9.59E-01 | 0.0 |
| Serum-TG | Serum total triglycerides | 0.051 | 0.013 | 1.31E-04 | 14.6 | -0.055 | 0.025 | 2.88E-02 | 4.8 |
| VLDL-TG | Triglycerides in VLDL | 0.052 | 0.013 | 5.35E-05 | 16.3 | -0.043 | 0.027 | 1.20E-01 | 2.4 |
| LDL-TG | Triglycerides in LDL | 0.030 | 0.010 | 2.07E-03 | 9.5 | -0.041 | 0.010 | 4.34E-05 | 16.7 |
| HDL-TG | Triglycerides in HDL | 0.053 | 0.019 | 5.04E-03 | 7.9 | -0.081 | 0.010 | 1.85E-15 | 63.2 |
| DAG | Diacylglycerol | 0.052 | 0.010 | 8.76E-07 | 24.2 | -0.067 | 0.014 | 8.75E-07 | 24.2 |
| DAG/TG | Ratio of diacylglycerol to triglycerides | 0.031 | 0.011 | 4.07E-03 | 8.3 | -0.046 | 0.011 | 2.48E-05 | 17.8 |
| TotPG | Total phosphoglycerides | 0.031 | 0.013 | 1.68E-02 | 5.7 | -0.042 | 0.014 | 2.38E-03 | 9.2 |
| TG/PG | Ratio of triglycerides to phosphoglycerides | 0.050 | 0.010 | 1.76E-07 | 27.3 | -0.041 | 0.030 | 1.67E-01 | 1.9 |
| PC | Phosphatidylcholine and other cholines | 0.031 | 0.014 | 2.82E-02 | 4.8 | -0.025 | 0.017 | 1.48E-01 | 2.1 |
| SM | Sphingomyelins | 0.009 | 0.009 | 3.60E-01 | 0.8 | 0.018 | 0.010 | 5.47E-02 | 3.7 |
| TotCho | Total cholines | 0.021 | 0.012 | 8.74E-02 | 2.9 | -0.024 | 0.016 | 1.31E-01 | 2.3 |
| ApoA1 | Apolipoprotein A-I | 0.010 | 0.009 | 2.77E-01 | 1.2 | -0.016 | 0.029 | 5.72E-01 | 0.3 |
| ApoB | Apolipoprotein B | 0.035 | 0.012 | 3.62E-03 | 8.5 | -0.040 | 0.018 | 2.69E-02 | 4.9 |
| ApoB/ApoA1 | Ratio of apolipoprotein B to apolipoprotein A-I | 0.031 | 0.012 | 1.18E-02 | 6.4 | -0.030 | 0.028 | 2.93E-01 | 1.1 |
| TotFA | Total fatty acids | 0.048 | 0.012 | 1.10E-04 | 15.0 | -0.044 | 0.014 | 1.79E-03 | 9.8 |
| FALen | Estimated description of fatty acid chain length. not actual carbon number | 0.019 | 0.011 | 7.92E-02 | 3.1 | -0.006 | 0.011 | 5.64E-01 | 0.3 |
| UnsatDeg | Estimated degree of unsaturation | -0.015 | 0.019 | 4.40E-01 | 0.6 | 0.034 | 0.027 | 2.11E-01 | 1.6 |
| DHA | 22:6. docosahexaenoic acid | 0.045 | 0.010 | 6.67E-06 | 20.3 | 0.020 | 0.016 | 2.10E-01 | 1.6 |
| LA | 18:2. linoleic acid | 0.033 | 0.012 | 6.58E-03 | 7.4 | -0.020 | 0.010 | 5.03E-02 | 3.8 |
| CLA | Conjugated linoleic acid | 0.033 | 0.024 | 1.62E-01 | 2.0 | -0.056 | 0.012 | 2.40E-06 | 22.3 |
| FAw3 | Omega-3 fatty acids | 0.043 | 0.010 | 1.08E-05 | 19.4 | -0.011 | 0.015 | 4.39E-01 | 0.6 |
| FAw6 | Omega-6 fatty acids | 0.035 | 0.011 | 1.88E-03 | 9.7 | -0.017 | 0.010 | 8.77E-02 | 2.9 |
|  |  | **TSH** | | | | **fT4** | | | |
| **Metabolic particle** | **Description** | **Pooled estimate** | **Pooled SE** | **P-value** | **I²** | **Pooled estimate** | **Pooled SE** | **P-value** | **I²** |
| PUFA | Polyunsaturated fatty acids | 0.035 | 0.012 | 3.35E-03 | 8.6 | -0.018 | 0.010 | 7.92E-02 | 3.1 |
| MUFA | Monounsaturated fatty acids; 16:1. 18:1 | 0.049 | 0.015 | 1.24E-03 | 10.4 | -0.043 | 0.018 | 1.73E-02 | 5.7 |
| SFA | Saturated fatty acids | 0.042 | 0.010 | 4.25E-05 | 16.8 | -0.064 | 0.010 | 1.83E-10 | 40.6 |
| DHA/FA | Ratio of 22:6 docosahexaenoic acid to total fatty acids | 0.019 | 0.013 | 1.30E-01 | 2.3 | 0.039 | 0.020 | 5.49E-02 | 3.7 |
| LA/FA | Ratio of 18:2 linoleic acid to total fatty acids | -0.025 | 0.013 | 5.73E-02 | 3.6 | -0.003 | 0.030 | 9.18E-01 | 0.0 |
| CLA/FA | Ratio of conjugated linoleic acid to total fatty acids | 0.017 | 0.025 | 4.99E-01 | 0.5 | -0.038 | 0.011 | 4.90E-04 | 12.2 |
| FAw3/FA | Ratio of omega-3 fatty acids to total fatty acids | -0.002 | 0.025 | 9.44E-01 | 0.0 | 0.026 | 0.013 | 5.31E-02 | 3.7 |
| FAw6/FA | Ratio of omega-6 fatty acids to total fatty acids | -0.034 | 0.013 | 9.91E-03 | 6.7 | 0.012 | 0.032 | 7.10E-01 | 0.1 |
| PUFA/FA | Ratio of polyunsaturated fatty acids to total fatty acids | -0.037 | 0.015 | 1.71E-02 | 5.7 | 0.022 | 0.031 | 4.69E-01 | 0.5 |
| MUFA/FA | Ratio of monounsaturated fatty acids to total fatty acids | 0.032 | 0.014 | 2.45E-02 | 5.1 | -0.010 | 0.020 | 6.35E-01 | 0.2 |
| SFA/FA | Ratio of saturated fatty acids to total fatty acids | -0.011 | 0.017 | 4.99E-01 | 0.5 | -0.042 | 0.024 | 7.87E-02 | 3.1 |
| Glc | Glucose | 0.029 | 0.022 | 1.82E-01 | 1.8 | -0.053 | 0.034 | 1.18E-01 | 2.5 |
| Lac | Lactate | 0.014 | 0.012 | 2.36E-01 | 1.4 | 0.040 | 0.015 | 8.09E-03 | 7.0 |
| Pyr | Pyruvate | 0.022 | 0.018 | 2.21E-01 | 1.5 | -0.001 | 0.025 | 9.70E-01 | 0.0 |
| Cit | Citrate | 0.036 | 0.010 | 3.62E-04 | 12.7 | 0.011 | 0.010 | 2.83E-01 | 1.2 |
| Ala | Alanine | 0.034 | 0.012 | 5.01E-03 | 7.9 | -0.011 | 0.017 | 5.12E-01 | 0.4 |
| Gln | Glutamine | -0.036 | 0.010 | 2.71E-04 | 13.3 | 0.000 | 0.018 | 9.82E-01 | 0.0 |
| His | Histidine | -0.003 | 0.010 | 7.68E-01 | 0.1 | -0.052 | 0.016 | 8.51E-04 | 11.1 |
| Ile | Isoleucine | 0.045 | 0.016 | 4.81E-03 | 8.0 | -0.075 | 0.030 | 1.12E-02 | 6.4 |
| Leu | Leucine | 0.024 | 0.018 | 1.65E-01 | 1.9 | -0.066 | 0.030 | 2.53E-02 | 5.0 |
| Val | Valine | 0.015 | 0.017 | 3.70E-01 | 0.8 | -0.063 | 0.032 | 4.72E-02 | 3.9 |
| Phe | Phenylalanine | 0.033 | 0.010 | 9.77E-04 | 10.9 | -0.059 | 0.024 | 1.40E-02 | 6.0 |
| Tyr | Tyrosine | 0.011 | 0.013 | 3.71E-01 | 0.8 | -0.045 | 0.012 | 1.71E-04 | 14.1 |
| Ace | Acetate | 0.013 | 0.010 | 2.08E-01 | 1.6 | -0.031 | 0.025 | 2.15E-01 | 1.5 |
| AcAce | Acetoacetate | 0.002 | 0.012 | 8.76E-01 | 0.0 | 0.063 | 0.017 | 3.16E-04 | 13.0 |
| bOHBut | 3-hydroxybutyrate | -0.007 | 0.012 | 5.41E-01 | 0.4 | 0.067 | 0.026 | 1.02E-02 | 6.6 |
| Crea | Creatinine | 0.078 | 0.009 | 1.17E-17 | 73.2 | 0.029 | 0.012 | 1.18E-02 | 6.3 |
|  |  | **TSH** | | | | **fT4** | | | |
| **Metabolic particle** | **Description** | **Pooled estimate** | **Pooled SE** | **P-value** | **I²** | **Pooled estimate** | **Pooled SE** | **P-value** | **I²** |
| Alb | Albumin | 0.060 | 0.010 | 2.40E-09 | 35.6 | 0.053 | 0.028 | 5.73E-02 | 3.6 |
| Gp | Glycoprotein acetyls. mainly a1-acid glycoprotein | 0.047 | 0.010 | 3.13E-06 | 21.7 | 0.036 | 0.029 | 2.09E-01 | 1.6 |

Abbreviations: TSH; thyroid stimulating hormone, fT4; free thyroxine, SE; standard error.

Pooled estimates, standard errors and p-values were derived from a random effects model meta-analysis of study-level multivariable regression adjusted for age, sex, body mass index and smoking status.

**Online Table 4. Second stage associations between metabolomic markers associated with TSH and fT4 in Mendelian randomization analyses and Bruker platform**

|  |  |  | **Mendelian randomization** | | | **Bruker platform** | | |
| --- | --- | --- | --- | --- | --- | --- | --- | --- |
| **Metabolic particle** | **Bruker name** | **Bruker description** | **Pooled estimate** | **Pooled SE** | **P-value** | **Estimate** | **SE** | **P-value** |
| **TSH results** |  |  |  |  |  |  |  |  |
| XXL-VLDL-P |  |  | 0.028 | 0.024 | 2.47E-01 | N.A. | N.A. | N.A. |
| XXL-VLDL-L |  |  | 0.023 | 0.023 | 3.32E-01 | N.A. | N.A. | N.A. |
| XXL-VLDL-PL | V1PL | VLDL1 Phospholipids (mg/dL) | 0.035 | 0.024 | 1.38E-01 | 0.077 | 0.031 | 1.35E-02 |
| XXL-VLDL-C | V1CH | VLDL1 Cholesterol (mg/dL) | N.A. | N.A. | N.A. | 0.089 | 0.032 | 5.65E-03 |
| XXL-VLDL-FC | V1FC | VLDL1 Free Cholesterol (mg/dL) | N.A. | N.A. | N.A. | 0.094 | 0.032 | 3.43E-03 |
| XXL-VLDL-TG | V1TG | VLDL1 Triglycerides (mg/dL) | 0.040 | 0.023 | 8.51E-02 | 0.080 | 0.031 | 9.79E-03 |
| XL-VLDL-P |  |  | 0.023 | 0.023 | 3.15E-01 | N.A. | N.A. | N.A. |
| XL-VLDL-L |  |  | 0.020 | 0.024 | 4.07E-01 | N.A. | N.A. | N.A. |
| XL-VLDL-C | V2CH | VLDL2 Cholesterol (mg/dL) | N.A. | N.A. | N.A. | 0.087 | 0.033 | 9.42E-03 |
| XL-VLDL-FC | V2FC | VLDL2 Free Cholesterol (mg/dL) | N.A. | N.A. | N.A. | 0.089 | 0.034 | 9.67E-03 |
| XL-VLDL-TG | V2TG | VLDL2 Triglycerides (mg/dL) | 0.027 | 0.024 | 2.65E-01 | 0.094 | 0.032 | 3.85E-03 |
| L-VLDL-P |  |  | 0.029 | 0.022 | 1.96E-01 | N.A. | N.A. | N.A. |
| L-VLDL-L |  |  | 0.026 | 0.023 | 2.61E-01 | N.A. | N.A. | N.A. |
| L-VLDL-PL | V3PL | VLDL3 Phospholipids (mg/dL) | 0.027 | 0.022 | 2.07E-01 | 0.079 | 0.034 | 1.85E-02 |
| L-VLDL-C | V3CH | VLDL3 Cholesterol (mg/dL) | 0.031 | 0.023 | 1.71E-01 | 0.080 | 0.034 | 1.85E-02 |
| L-VLDL-CE |  |  | 0.031 | 0.022 | 1.56E-01 | N.A. | N.A. | N.A. |
| L-VLDL-FC | V3FC | VLDL3 Free Cholesterol (mg/dL) | 0.030 | 0.023 | 1.89E-01 | 0.084 | 0.034 | 1.25E-02 |
| L-VLDL-TG | V3TG | VLDL3 Triglycerides (mg/dL) | 0.037 | 0.022 | 9.54E-02 | 0.080 | 0.033 | 1.76E-02 |
| M-VLDL-P |  |  | 0.031 | 0.023 | 1.72E-01 | N.A. | N.A. | N.A. |
| M-VLDL-L |  |  | 0.038 | 0.023 | 9.25E-02 | N.A. | N.A. | N.A. |
| M-VLDL-PL | V4PL | VLDL4 Phospholipids (mg/dL) | 0.041 | 0.022 | 6.46E-02 | 0.060 | 0.034 | 7.47E-02 |
| M-VLDL-C | V4CH | VLDL4 Cholesterol (mg/dL) | 0.042 | 0.021 | 5.28E-02 | 0.052 | 0.034 | 1.30E-01 |
| M-VLDL-CE |  |  | 0.038 | 0.022 | 8.26E-02 | N.A. | N.A. | N.A. |
| M-VLDL-FC | V4FC | VLDL4 Free Cholesterol (mg/dL) | 0.044 | 0.023 | 5.19E-02 | 0.066 | 0.034 | 5.35E-02 |
|  |  |  | **Mendelian randomization** | | | **Bruker platform** | | |
| **Metabolic particle** | **Bruker name** | **Bruker description** | **Pooled estimate** | **Pooled SE** | **P-value** | **Estimate** | **SE** | **P-value** |
| **TSH results** |  |  |  |  |  |  |  |  |
| M-VLDL-TG | V4TG | VLDL4 Triglycerides (mg/dL) | 0.042 | 0.022 | 6.00E-02 | 0.057 | 0.034 | 9.29E-02 |
| S-VLDL-P |  |  | 0.045 | 0.022 | 3.97E-02 | N.A. | N.A. | N.A. |
| S-VLDL-L |  |  | 0.048 | 0.022 | 2.93E-02 | N.A. | N.A. | N.A. |
| S-VLDL-PL | V5PL | VLDL5 Phospholipids (mg/dL) | 0.051 | 0.021 | 1.76E-02 | 0.059 | 0.036 | 1.01E-01 |
| S-VLDL-FC | V5FC | VLDL5 Free Cholesterol (mg/dL) | 0.045 | 0.021 | 3.77E-02 | 0.026 | 0.047 | 5.80E-01 |
| S-VLDL-TG | V5TG | VLDL5 Triglycerides (mg/dL) | 0.043 | 0.022 | 5.47E-02 | 0.041 | 0.034 | 2.29E-01 |
| XS-VLDL-TG | V6TG | VLDL6 Triglycerides (mg/dL) | 0.031 | 0.022 | 1.60E-01 | 0.020 | 0.036 | 5.74E-01 |
| IDL-TG | IDTG | IDL Triglycerides (mg/dL) | 0.008 | 0.022 | 7.08E-01 | 0.084 | 0.033 | 1.18E-02 |
| S-HDL-TG | H4TG | HDL4 Triglycerides (mg/dL) | 0.000 | 0.022 | 9.87E-01 | 0.058 | 0.035 | 9.29E-02 |
| VLDL-D |  |  | 0.030 | 0.023 | 1.99E-01 | N.A. | N.A. | N.A. |
| Serum-TG | TPTG | Total Triglycerides (mg/dL) | 0.041 | 0.022 | 6.54E-02 | 0.095 | 0.032 | 2.75E-03 |
| VLDL-TG | VLTG | VLDL Triglycerides (mg/dL) | N.A. | N.A. | N.A. | 0.087 | 0.032 | 5.89E-03 |
| TotFA |  |  | 0.055 | 0.024 | 2.24E-02 | N.A. | N.A. | N.A. |
| DHA |  |  | 0.023 | 0.026 | 3.74E-01 | N.A. | N.A. | N.A. |
| FAw3 |  |  | 0.038 | 0.026 | 1.44E-01 | N.A. | N.A. | N.A. |
| MUFA |  |  | 0.036 | 0.024 | 1.34E-01 | N.A. | N.A. | N.A. |
| Cit |  |  | -0.032 | 0.021 | 1.31E-01 | N.A. | N.A. | N.A. |
| Gln |  |  | 0.042 | 0.023 | 5.93E-02 | N.A. | N.A. | N.A. |
| Phe |  |  | -0.029 | 0.022 | 1.84E-01 | N.A. | N.A. | N.A. |
| Crea |  |  | 0.046 | 0.024 | 5.20E-02 | N.A. | N.A. | N.A. |
| Alb |  |  | -0.001 | 0.022 | 9.49E-01 | N.A. | N.A. | N.A. |
| Gp |  |  | -0.013 | 0.023 | 5.71E-01 | N.A. | N.A. | N.A. |
| **fT4 results** |  |  |  |  |  |  |  |  |
| XXL-VLDL-C | V1CH | VLDL1 Cholesterol (mg/dL) | N.A. | N.A. | N.A. | -0.056 | 0.032 | 7.57E-02 |
| XXL-VLDL-FC | V1FC | VLDL1 Free Cholesterol (mg/dL) | N.A. | N.A. | N.A. | -0.052 | 0.032 | 1.03E-01 |
| XL-VLDL-C | V2CH | VLDL2 Cholesterol (mg/dL) | N.A. | N.A. | N.A. | -0.062 | 0.033 | 5.77E-02 |
|  |  |  | **Mendelian randomization** | | | **Bruker platform** | | |
| **Metabolic particle** | **Bruker name** | **Bruker description** | **Pooled estimate** | **Pooled SE** | **P-value** | **Estimate** | **SE** | **P-value** |
| **fT4 results** |  |  |  |  |  |  |  |  |
| XL-VLDL-FC | V2FC | VLDL2 Free Cholesterol (mg/dL) | N.A. | N.A. | N.A. | -0.057 | 0.033 | 8.60E-02 |
| IDL-TG | IDTG | IDL Triglycerides (mg/dL) | -0.012 | 0.032 | 6.97E-01 | -0.081 | 0.032 | 1.26E-02 |
| M-HDL-TG | H3TG | HDL3 Triglycerides (mg/dL) | N.A. | N.A. | N.A. | -0.094 | 0.034 | 6.15E-03 |
| S-HDL-PL | H4PL | HDL4 Phospholipids (mg/dL) | N.A. | N.A. | N.A. | -0.111 | 0.036 | 1.85E-03 |
| LDL-TG | LDTG | LDL Triglycerides (mg/dL) | N.A. | N.A. | N.A. | -0.064 | 0.032 | 4.75E-02 |
| HDL-TG | HDTG | HDL Triglycerides (mg/dL) | N.A. | N.A. | N.A. | -0.094 | 0.034 | 5.30E-03 |
| His |  |  | -0.009 | 0.035 | 7.99E-01 | N.A. | N.A. | N.A. |
| Tyr |  |  | 0.014 | 0.035 | 6.86E-01 | N.A. | N.A. | N.A. |
| AcAce |  |  | 0.044 | 0.033 | 1.81E-01 | N.A. | N.A. | N.A. |

Abbreviations: TSH; thyroid stimulating hormone, fT4; free thyroxine, SE; standard error, N.A.; not available.

Pooled estimates, standard errors and p-values for Mendelian randomization were derived from a fixed effects model meta-analysis of study-level inverse-variance weighted analysis.

Effect estimates, standard errors and p-values for the Bruker platform were derived from a single study multivariable regression analysis adjusted for age, sex, body mass index and smoking status.

**Online Table 5. Sensitivity analyses for metabolomic markers associated with TSH and fT4 in a restricted population without thyroid medication, lipid-lowering medication or history of diabetes**

| **Metabolic particle** | **Pooled estimate** | **Pooled SE** | **P-value** | **I²** |
| --- | --- | --- | --- | --- |
| **TSH results** |  |  |  |  |
| XXL-VLDL-P | 0.041 | 0.019 | 3.29E-02 | 4.55 |
| XXL-VLDL-L | 0.040 | 0.020 | 4.63E-02 | 3.97 |
| XXL-VLDL-PL | 0.041 | 0.020 | 4.75E-02 | 3.93 |
| XXL-VLDL-C | 0.038 | 0.019 | 4.39E-02 | 4.06 |
| XXL-VLDL-CE | 0.036 | 0.017 | 3.77E-02 | 4.32 |
| XXL-VLDL-FC | 0.042 | 0.020 | 3.21E-02 | 4.59 |
| XXL-VLDL-TG | 0.041 | 0.020 | 3.71E-02 | 4.35 |
| XL-VLDL-P | 0.041 | 0.020 | 3.71E-02 | 4.34 |
| XL-VLDL-L | 0.037 | 0.021 | 7.65E-02 | 3.14 |
| XL-VLDL-C | 0.037 | 0.020 | 5.85E-02 | 3.58 |
| XL-VLDL-CE | 0.037 | 0.019 | 5.17E-02 | 3.79 |
| XL-VLDL-FC | 0.041 | 0.019 | 3.72E-02 | 4.34 |
| XL-VLDL-TG | 0.040 | 0.020 | 5.01E-02 | 3.84 |
| L-VLDL-P | 0.040 | 0.021 | 5.14E-02 | 3.80 |
| L-VLDL-L | 0.032 | 0.023 | 1.61E-01 | 1.97 |
| L-VLDL-PL | 0.038 | 0.021 | 7.38E-02 | 3.20 |
| L-VLDL-C | 0.037 | 0.021 | 7.73E-02 | 3.12 |
| L-VLDL-CE | 0.037 | 0.019 | 4.33E-02 | 4.08 |
| L-VLDL-FC | 0.039 | 0.022 | 7.09E-02 | 3.26 |
| L-VLDL-TG | 0.036 | 0.022 | 1.03E-01 | 2.65 |
| M-VLDL-P | 0.039 | 0.019 | 4.12E-02 | 4.17 |
| M-VLDL-L | 0.032 | 0.021 | 1.36E-01 | 2.22 |
| M-VLDL-PL | 0.038 | 0.019 | 4.58E-02 | 3.99 |
| M-VLDL-C | 0.033 | 0.020 | 1.03E-01 | 2.66 |
| M-VLDL-CE | 0.031 | 0.018 | 8.76E-02 | 2.92 |
| M-VLDL-FC | 0.037 | 0.021 | 8.23E-02 | 3.02 |
| M-VLDL-TG | 0.036 | 0.020 | 7.61E-02 | 3.15 |
| S-VLDL-P | 0.030 | 0.018 | 8.71E-02 | 2.93 |
| S-VLDL-L | 0.024 | 0.018 | 1.95E-01 | 1.68 |
| S-VLDL-PL | 0.020 | 0.020 | 3.22E-01 | 0.98 |
| S-VLDL-FC | 0.028 | 0.017 | 1.04E-01 | 2.65 |
| S-VLDL-TG | 0.031 | 0.019 | 9.84E-02 | 2.73 |
| XS-VLDL-TG | 0.025 | 0.016 | 1.20E-01 | 2.41 |
| IDL-TG | 0.006 | 0.017 | 7.34E-01 | 0.12 |
| S-LDL-TG | 0.010 | 0.023 | 6.45E-01 | 0.21 |
| S-HDL-TG | 0.022 | 0.023 | 3.41E-01 | 0.91 |
| VLDL-D | 0.038 | 0.022 | 8.49E-02 | 2.97 |
| Serum-TG | 0.023 | 0.023 | 3.32E-01 | 0.94 |
| VLDL-TG | 0.033 | 0.021 | 1.08E-01 | 2.59 |
| DAG | 0.033 | 0.018 | 6.90E-02 | 3.31 |
| TG/PG | 0.024 | 0.018 | 1.79E-01 | 1.81 |
| **Metabolic particle** | **Pooled estimate** | **Pooled SE** | **P-value** | **I²** |
| **TSH results** |  |  |  |  |
| TotFA | 0.021 | 0.023 | 3.76E-01 | 0.78 |
| DHA | 0.038 | 0.013 | 3.86E-03 | 8.35 |
| FAw3 | 0.034 | 0.013 | 8.05E-03 | 7.02 |
| MUFA | 0.010 | 0.029 | 7.20E-01 | 0.13 |
| SFA | 0.022 | 0.024 | 3.60E-01 | 0.84 |
| Cit | 0.028 | 0.013 | 3.27E-02 | 4.56 |
| Gln | -0.032 | 0.013 | 1.56E-02 | 5.85 |
| Phe | 0.034 | 0.013 | 1.16E-02 | 6.37 |
| Crea | 0.073 | 0.012 | 3.33E-10 | 39.47 |
| Alb | 0.064 | 0.013 | 1.19E-06 | 23.60 |
| Gp | 0.037 | 0.017 | 2.90E-02 | 4.77 |
| **fT4 results** |  |  |  |  |
| XXL-VLDL-C | -0.058 | 0.025 | 1.74E-02 | 5.66 |
| XXL-VLDL-CE | -0.057 | 0.023 | 1.26E-02 | 6.23 |
| XXL-VLDL-FC | -0.061 | 0.025 | 1.69E-02 | 5.71 |
| XL-VLDL-C | -0.060 | 0.025 | 1.76E-02 | 5.64 |
| XL-VLDL-CE | -0.061 | 0.025 | 1.26E-02 | 6.23 |
| XL-VLDL-FC | -0.057 | 0.026 | 2.62E-02 | 4.94 |
| IDL-TG | -0.051 | 0.016 | 1.12E-03 | 10.61 |
| L-LDL-TG | -0.052 | 0.013 | 6.49E-05 | 15.95 |
| S-LDL-TG | -0.062 | 0.013 | 2.11E-06 | 22.49 |
| M-HDL-TG | -0.081 | 0.019 | 2.13E-05 | 18.07 |
| S-HDL-PL | -0.039 | 0.024 | 1.08E-01 | 2.59 |
| LDL-TG | -0.051 | 0.013 | 8.53E-05 | 15.44 |
| HDL-TG | -0.094 | 0.013 | 3.50E-12 | 48.38 |
| DAG | -0.075 | 0.023 | 1.10E-03 | 10.65 |
| DAG/TG | -0.051 | 0.014 | 3.57E-04 | 12.75 |
| CLA | -0.062 | 0.020 | 1.72E-03 | 9.83 |
| SFA | -0.057 | 0.016 | 3.34E-04 | 12.87 |
| CLA/FA | -0.045 | 0.019 | 1.73E-02 | 5.66 |
| His | -0.052 | 0.016 | 1.02E-03 | 10.79 |
| Tyr | -0.037 | 0.017 | 3.09E-02 | 4.66 |
| AcAce | 0.080 | 0.015 | 2.47E-07 | 26.63 |

Abbreviations: TSH; thyroid stimulating hormone, fT4; free thyroxine, SE; standard error, N.A.; not available.

Pooled estimates, standard errors and p-values were derived from a random effects model meta-analysis of study-level multivariable regression adjusted for age, sex, body mass index and smoking status excluding all participants who used thyroid therapy or lipid-lowering medication or had diabetes mellitus.

**Online Table 6. Sensitivity analyses for Mendelian randomization analyses of metabolomic markers and TSH and fT4**

|  |  | **MR Egger** | | | **WME** | | | **MR Bruker platform** | | |
| --- | --- | --- | --- | --- | --- | --- | --- | --- | --- | --- |
| **Metabolic particle** | **Bruker name** | **Pooled estimate** | **Pooled SE** | **P-value** | **Pooled estimate** | **Pooled SE** | **P-value** | **Estimate** | **SE** | **P-value** |
| **TSH results** |  |  |  |  |  |  |  |  |  |  |
| XXL-VLDL-P |  | 0.033 | 0.061 | 5.84E-01 | 0.022 | 0.033 | 5.03E-01 | N.A. | N.A. | N.A. |
| XXL-VLDL-L |  | 0.004 | 0.058 | 9.39E-01 | 0.019 | 0.032 | 5.52E-01 | N.A. | N.A. | N.A. |
| XXL-VLDL-PL | V1PL | 0.027 | 0.059 | 6.41E-01 | 0.036 | 0.033 | 2.79E-01 | -0.036 | 0.103 | 7.28E-01 |
| XXL-VLDL-C | V1CH | N.A. | N.A. | N.A. | N.A. | N.A. | N.A. | 0.037 | 0.103 | 7.20E-01 |
| XXL-VLDL-FC | V1FC | N.A. | N.A. | N.A. | N.A. | N.A. | N.A. | -0.017 | 0.092 | 8.52E-01 |
| XXL-VLDL-TG | V1TG | 0.049 | 0.058 | 4.01E-01 | 0.031 | 0.032 | 3.23E-01 | -0.040 | 0.113 | 7.21E-01 |
| XL-VLDL-P |  | 0.003 | 0.056 | 9.57E-01 | 0.022 | 0.033 | 5.00E-01 | N.A. | N.A. | N.A. |
| XL-VLDL-L |  | 0.005 | 0.059 | 9.26E-01 | 0.019 | 0.034 | 5.89E-01 | N.A. | N.A. | N.A. |
| XL-VLDL-C | V2CH | N.A. | N.A. | N.A. | N.A. | N.A. | N.A. | 0.047 | 0.112 | 6.75E-01 |
| XL-VLDL-FC | V2FC | N.A. | N.A. | N.A. | N.A. | N.A. | N.A. | 0.040 | 0.098 | 6.86E-01 |
| XL-VLDL-TG | V2TG | 0.007 | 0.057 | 9.08E-01 | 0.022 | 0.032 | 4.88E-01 | 0.031 | 0.112 | 7.78E-01 |
| L-VLDL-P |  | 0.028 | 0.055 | 6.16E-01 | 0.014 | 0.033 | 6.66E-01 | N.A. | N.A. | N.A. |
| L-VLDL-L |  | 0.010 | 0.058 | 8.62E-01 | 0.016 | 0.033 | 6.32E-01 | N.A. | N.A. | N.A. |
| L-VLDL-PL | V3PL | 0.027 | 0.054 | 6.17E-01 | 0.024 | 0.032 | 4.50E-01 | 0.015 | 0.111 | 8.91E-01 |
| L-VLDL-C | V3CH | 0.043 | 0.057 | 4.54E-01 | 0.022 | 0.033 | 5.04E-01 | 0.029 | 0.120 | 8.07E-01 |
| L-VLDL-CE |  | 0.046 | 0.055 | 3.97E-01 | 0.058 | 0.033 | 7.68E-02 | N.A. | N.A. | N.A. |
| L-VLDL-FC | V3FC | 0.026 | 0.057 | 6.50E-01 | 0.034 | 0.032 | 2.93E-01 | 0.037 | 0.103 | 7.19E-01 |
| L-VLDL-TG | V3TG | 0.037 | 0.056 | 5.07E-01 | 0.023 | 0.033 | 4.85E-01 | 0.050 | 0.113 | 6.57E-01 |
| M-VLDL-P |  | -0.015 | 0.056 | 7.88E-01 | 0.029 | 0.033 | 3.80E-01 | N.A. | N.A. | N.A. |
| M-VLDL-L |  | 0.006 | 0.057 | 9.22E-01 | 0.030 | 0.033 | 3.69E-01 | N.A. | N.A. | N.A. |
| M-VLDL-PL | V4PL | 0.021 | 0.055 | 6.96E-01 | 0.041 | 0.033 | 2.14E-01 | -0.011 | 0.132 | 9.34E-01 |
| M-VLDL-C | V4CH | 0.021 | 0.053 | 6.93E-01 | 0.013 | 0.032 | 6.85E-01 | -0.014 | 0.132 | 9.18E-01 |
| M-VLDL-CE |  | -0.001 | 0.054 | 9.91E-01 | 0.015 | 0.034 | 6.56E-01 | N.A. | N.A. | N.A. |
| M-VLDL-FC | V4FC | 0.022 | 0.056 | 6.99E-01 | 0.034 | 0.032 | 2.82E-01 | -0.008 | 0.132 | 9.54E-01 |
|  |  |  |  |  |  |  |  |  |  |  |
|  |  | **MR Egger** | | | **WME** | | | **MR Bruker platform** | | |
| **Metabolic particle** | **Bruker name** | **Pooled estimate** | **Pooled SE** | **P-value** | **Pooled estimate** | **Pooled SE** | **P-value** | **Estimate** | **SE** | **P-value** |
| **TSH results** |  |  |  |  |  |  |  |  |  |  |
| M-VLDL-TG | V4TG | 0.028 | 0.056 | 6.15E-01 | 0.044 | 0.032 | 1.72E-01 | 0.035 | 0.133 | 7.92E-01 |
| S-VLDL-P |  | -0.001 | 0.054 | 9.86E-01 | 0.032 | 0.033 | 3.36E-01 | N.A. | N.A. | N.A. |
| S-VLDL-L |  | -0.001 | 0.054 | 9.80E-01 | 0.028 | 0.033 | 3.96E-01 | N.A. | N.A. | N.A. |
| S-VLDL-PL | V5PL | 0.008 | 0.053 | 8.77E-01 | 0.023 | 0.032 | 4.61E-01 | -0.062 | 0.139 | 6.54E-01 |
| S-VLDL-FC | V5FC | 0.003 | 0.054 | 9.51E-01 | 0.011 | 0.033 | 7.46E-01 | 0.120 | 0.150 | 4.22E-01 |
| S-VLDL-TG | V5TG | 0.024 | 0.055 | 6.59E-01 | 0.015 | 0.033 | 6.45E-01 | 0.066 | 0.136 | 6.28E-01 |
| XS-VLDL-TG | V6TG | 0.008 | 0.061 | 8.90E-01 | 0.027 | 0.033 | 4.04E-01 | 0.164 | 0.123 | 1.81E-01 |
| IDL-TG | IDTG | -0.044 | 0.054 | 4.13E-01 | 0.015 | 0.031 | 6.35E-01 | 0.105 | 0.118 | 3.77E-01 |
| S-HDL-TG | H4TG | -0.015 | 0.055 | 7.82E-01 | -0.009 | 0.031 | 7.70E-01 | 0.027 | 0.144 | 8.51E-01 |
| VLDL-D |  | 0.003 | 0.057 | 9.53E-01 | 0.018 | 0.033 | 5.89E-01 | N.A. | N.A. | N.A. |
| Serum-TG | TPTG | 0.037 | 0.055 | 5.03E-01 | 0.027 | 0.033 | 4.16E-01 | 0.045 | 0.109 | 6.82E-01 |
| VLDL-TG | VLTG | N.A. | N.A. | N.A. | N.A. | N.A. | N.A. | 0.040 | 0.102 | 6.98E-01 |
| TotFA |  | -0.009 | 0.059 | 8.85E-01 | 0.064 | 0.036 | 7.56E-02 | N.A. | N.A. | N.A. |
| DHA |  | 0.060 | 0.063 | 3.37E-01 | 0.036 | 0.036 | 3.21E-01 | N.A. | N.A. | N.A. |
| FAw3 |  | 0.034 | 0.064 | 5.96E-01 | 0.028 | 0.038 | 4.61E-01 | N.A. | N.A. | N.A. |
| MUFA |  | -0.025 | 0.059 | 6.69E-01 | 0.025 | 0.036 | 4.96E-01 | N.A. | N.A. | N.A. |
| Cit |  | -0.063 | 0.052 | 2.29E-01 | -0.057 | 0.030 | 6.04E-02 | N.A. | N.A. | N.A. |
| Gln |  | -0.080 | 0.055 | 1.43E-01 | 0.001 | 0.031 | 9.62E-01 | N.A. | N.A. | N.A. |
| Phe |  | -0.106 | 0.053 | 4.75E-02 | -0.049 | 0.031 | 1.13E-01 | N.A. | N.A. | N.A. |
| Crea |  | 0.008 | 0.058 | 8.93E-01 | 0.019 | 0.032 | 5.54E-01 | N.A. | N.A. | N.A. |
| Alb |  | -0.020 | 0.056 | 7.14E-01 | -0.018 | 0.033 | 5.75E-01 | N.A. | N.A. | N.A. |
| Gp |  | -0.008 | 0.057 | 8.93E-01 | -0.005 | 0.033 | 8.87E-01 | N.A. | N.A. | N.A. |
| **fT4 results** |  |  |  |  |  |  |  |  |  |  |
| XXL-VLDL-C | V1CH | N.A. | N.A. | N.A. | N.A. | N.A. | N.A. | 0.247 | 0.166 | 1.36E-01 |
| XXL-VLDL-CE |  | N.A. | N.A. | N.A. | N.A. | N.A. | N.A. | N.A. | N.A. | N.A. |
| XXL-VLDL-FC | V1FC | N.A. | N.A. | N.A. | N.A. | N.A. | N.A. | 0.158 | 0.161 | 3.25E-01 |
| XL-VLDL-C | V2CH | N.A. | N.A. | N.A. | N.A. | N.A. | N.A. | 0.216 | 0.148 | 1.43E-01 |
|  |  | **MR Egger** | | | **WME** | | | **MR Bruker platform** | | |
| **Metabolic particle** | **Bruker name** | **Pooled estimate** | **Pooled SE** | **P-value** | **Pooled estimate** | **Pooled SE** | **P-value** | **Estimate** | **SE** | **P-value** |
| **fT4 results** |  |  |  |  |  |  |  |  |  |  |
| XL-VLDL-FC | V2FC | N.A. | N.A. | N.A. | N.A. | N.A. | N.A. | 0.213 | 0.144 | 1.40E-01 |
| IDL-TG | IDTG | -0.008 | 0.076 | 9.13E-01 | 0.000 | 0.043 | 9.93E-01 | 0.311 | 0.176 | 7.72E-02 |
| M-HDL-TG | H3TG | N.A. | N.A. | N.A. | N.A. | N.A. | N.A. | 0.169 | 0.207 | 4.13E-01 |
| S-HDL-PL | H4PL | N.A. | N.A. | N.A. | N.A. | N.A. | N.A. | -0.131 | 0.207 | 5.26E-01 |
| LDL-TG | LDTG | N.A. | N.A. | N.A. | N.A. | N.A. | N.A. | 0.180 | 0.182 | 3.22E-01 |
| HDL-TG | HDTG | N.A. | N.A. | N.A. | N.A. | N.A. | N.A. | 0.048 | 0.209 | 8.20E-01 |
| His |  | -0.109 | 0.081 | 1.79E-01 | -0.080 | 0.042 | 5.65E-02 | N.A. | N.A. | N.A. |
| Tyr |  | 0.057 | 0.085 | 5.03E-01 | 0.031 | 0.041 | 4.45E-01 | N.A. | N.A. | N.A. |
| AcAce |  | -0.093 | 0.078 | 2.30E-01 | 0.008 | 0.046 | 8.69E-01 | N.A. | N.A. | N.A. |

Abbreviations: TSH; thyroid stimulating hormone, fT4; free thyroxine, SE; standard error, MR; Mendelian randomization; WME; Weighted Median Estimator, N.A.; not available.

Pooled estimates, standard errors and p-values for Mendelian randomization sensitivity analyses MR Egger and WME analyses were derived from a fixed effects model meta-analysis of study-level analyses.

Effect estimates, standard errors and p-values for Mendelian randomization on the Bruker platform were derived from a single study inverse-variance weighted analysis.

**Online Table 7. Associations between metabolomic markers associated with TSH and fT4 and biochemical thyroid dysfunction**

|  | **Hypothyroidism** | | | | **Hyperthyroidism** | | | |
| --- | --- | --- | --- | --- | --- | --- | --- | --- |
| **Metabolic particle** | **Pooled estimate** | **Pooled SE** | **P-value** | **I²** | **Pooled estimate** | **Pooled SE** | **P-value** | **I²** |
| **TSH results** |  |  |  |  |  |  |  |  |
| XXL-VLDL-P | 0.373 | 0.147 | 1.14E-02 | 6.40 | -0.157 | 0.135 | 2.43E-01 | 1.36 |
| XXL-VLDL-L | 0.376 | 0.147 | 1.04E-02 | 6.56 | -0.157 | 0.136 | 2.47E-01 | 1.34 |
| XXL-VLDL-PL | 0.359 | 0.147 | 1.46E-02 | 5.96 | -0.162 | 0.137 | 2.38E-01 | 1.39 |
| XXL-VLDL-C | 0.403 | 0.155 | 9.20E-03 | 6.78 | -0.139 | 0.133 | 2.95E-01 | 1.10 |
| XXL-VLDL-CE | 0.422 | 0.158 | 7.64E-03 | 7.12 | -0.126 | 0.133 | 3.46E-01 | 0.89 |
| XXL-VLDL-FC | 0.420 | 0.175 | 1.63E-02 | 5.77 | -0.174 | 0.132 | 1.88E-01 | 1.73 |
| XXL-VLDL-TG | 0.385 | 0.153 | 1.20E-02 | 6.32 | -0.156 | 0.142 | 2.72E-01 | 1.21 |
| XL-VLDL-P | 0.403 | 0.166 | 1.49E-02 | 5.93 | -0.181 | 0.143 | 2.06E-01 | 1.60 |
| XL-VLDL-L | 0.427 | 0.172 | 1.27E-02 | 6.21 | -0.176 | 0.147 | 2.32E-01 | 1.43 |
| XL-VLDL-C | 0.441 | 0.171 | 9.89E-03 | 6.65 | -0.153 | 0.132 | 2.47E-01 | 1.34 |
| XL-VLDL-CE | 0.455 | 0.175 | 9.44E-03 | 6.74 | -0.157 | 0.132 | 2.35E-01 | 1.41 |
| XL-VLDL-FC | 0.443 | 0.177 | 1.24E-02 | 6.25 | -0.147 | 0.132 | 2.66E-01 | 1.24 |
| XL-VLDL-TG | 0.444 | 0.183 | 1.52E-02 | 5.89 | -0.200 | 0.150 | 1.82E-01 | 1.78 |
| L-VLDL-P | 0.388 | 0.163 | 1.71E-02 | 5.69 | -0.171 | 0.137 | 2.12E-01 | 1.56 |
| L-VLDL-L | 0.399 | 0.160 | 1.29E-02 | 6.18 | -0.193 | 0.140 | 1.68E-01 | 1.90 |
| L-VLDL-PL | 0.437 | 0.181 | 1.58E-02 | 5.83 | -0.178 | 0.133 | 1.80E-01 | 1.80 |
| L-VLDL-C | 0.430 | 0.170 | 1.16E-02 | 6.37 | -0.185 | 0.132 | 1.59E-01 | 1.99 |
| L-VLDL-CE | 0.445 | 0.173 | 1.00E-02 | 6.63 | -0.160 | 0.132 | 2.26E-01 | 1.46 |
| L-VLDL-FC | 0.428 | 0.180 | 1.73E-02 | 5.67 | -0.204 | 0.132 | 1.21E-01 | 2.41 |
| L-VLDL-TG | 0.397 | 0.167 | 1.78E-02 | 5.62 | -0.190 | 0.140 | 1.73E-01 | 1.85 |
| M-VLDL-P | 0.417 | 0.174 | 1.62E-02 | 5.78 | -0.159 | 0.131 | 2.27E-01 | 1.46 |
| M-VLDL-L | 0.422 | 0.169 | 1.24E-02 | 6.26 | -0.189 | 0.131 | 1.47E-01 | 2.10 |
| M-VLDL-PL | 0.440 | 0.184 | 1.64E-02 | 5.75 | -0.170 | 0.131 | 1.93E-01 | 1.69 |
| M-VLDL-C | 0.457 | 0.172 | 7.96E-03 | 7.04 | -0.155 | 0.132 | 2.39E-01 | 1.38 |
|  | **Hypothyroidism** | | | | **Hyperthyroidism** | | | |
| **Metabolic particle** | **Pooled estimate** | **Pooled SE** | **P-value** | **I²** | **Pooled estimate** | **Pooled SE** | **P-value** | **I²** |
| **TSH results** |  |  |  |  |  |  |  |  |
| M-VLDL-CE | 0.452 | 0.164 | 6.01E-03 | 7.55 | -0.123 | 0.133 | 3.55E-01 | 0.85 |
| M-VLDL-FC | 0.456 | 0.187 | 1.46E-02 | 5.97 | -0.209 | 0.132 | 1.13E-01 | 2.51 |
| M-VLDL-TG | 0.427 | 0.184 | 2.05E-02 | 5.37 | -0.192 | 0.130 | 1.41E-01 | 2.17 |
| S-VLDL-P | 0.440 | 0.171 | 1.03E-02 | 6.59 | -0.164 | 0.131 | 2.11E-01 | 1.57 |
| S-VLDL-L | 0.472 | 0.191 | 1.34E-02 | 6.11 | -0.196 | 0.131 | 1.34E-01 | 2.25 |
| S-VLDL-PL | 0.444 | 0.193 | 2.17E-02 | 5.27 | -0.221 | 0.131 | 9.18E-02 | 2.84 |
| S-VLDL-FC | 0.412 | 0.163 | 1.15E-02 | 6.38 | -0.217 | 0.132 | 9.99E-02 | 2.71 |
| S-VLDL-TG | 0.503 | 0.209 | 1.63E-02 | 5.77 | -0.219 | 0.131 | 9.51E-02 | 2.79 |
| XS-VLDL-TG | 0.377 | 0.133 | 4.54E-03 | 8.05 | -0.245 | 0.132 | 6.32E-02 | 3.45 |
| IDL-TG | 0.323 | 0.122 | 8.26E-03 | 6.98 | -0.267 | 0.164 | 1.04E-01 | 2.64 |
| S-LDL-TG | 0.320 | 0.130 | 1.39E-02 | 6.05 | -0.280 | 0.131 | 3.28E-02 | 4.55 |
| S-HDL-TG | 0.293 | 0.114 | 9.79E-03 | 6.67 | -0.167 | 0.132 | 2.06E-01 | 1.60 |
| VLDL-D | 0.347 | 0.158 | 2.76E-02 | 4.85 | -0.248 | 0.155 | 1.10E-01 | 2.56 |
| Serum-TG | 0.303 | 0.121 | 1.25E-02 | 6.24 | -0.291 | 0.130 | 2.58E-02 | 4.97 |
| VLDL-TG | 0.442 | 0.181 | 1.45E-02 | 5.97 | -0.228 | 0.130 | 7.99E-02 | 3.07 |
| DAG | 0.075 | 0.077 | 3.30E-01 | 0.95 | -0.078 | 0.138 | 5.73E-01 | 0.32 |
| TG/PG | 0.267 | 0.084 | 1.46E-03 | 10.12 | -0.191 | 0.129 | 1.37E-01 | 2.22 |
| TotFA | 0.345 | 0.158 | 2.92E-02 | 4.76 | -0.304 | 0.131 | 1.98E-02 | 5.43 |
| DHA | -0.170 | 0.136 | 2.11E-01 | 1.57 | 0.036 | 0.134 | 7.87E-01 | 0.07 |
| FAw3 | 0.026 | 0.105 | 8.04E-01 | 0.06 | -0.173 | 0.133 | 1.91E-01 | 1.71 |
| MUFA | 0.242 | 0.131 | 6.43E-02 | 3.42 | -0.274 | 0.130 | 3.57E-02 | 4.41 |
| SFA | 0.353 | 0.151 | 1.91E-02 | 5.49 | -0.395 | 0.132 | 2.84E-03 | 8.91 |
| Cit | 0.073 | 0.070 | 3.00E-01 | 1.07 | -0.005 | 0.221 | 9.82E-01 | 0.00 |
| Gln | 0.080 | 0.071 | 2.61E-01 | 1.26 | 0.238 | 0.134 | 7.51E-02 | 3.17 |
| Phe | 0.123 | 0.070 | 7.86E-02 | 3.09 | 0.224 | 0.131 | 8.73E-02 | 2.92 |
| Crea | 0.133 | 0.064 | 3.81E-02 | 4.30 | 0.173 | 0.162 | 2.85E-01 | 1.14 |
|  | **Hypothyroidism** | | | | **Hyperthyroidism** | | | |
| **Metabolic particle** | **Pooled estimate** | **Pooled SE** | **P-value** | **I²** | **Pooled estimate** | **Pooled SE** | **P-value** | **I²** |
| **TSH results** |  |  |  |  |  |  |  |  |
| Alb | 0.162 | 0.109 | 1.37E-01 | 2.21 | -0.039 | 0.239 | 8.69E-01 | 0.03 |
| Gp | 0.172 | 0.070 | 1.38E-02 | 6.07 | 0.058 | 0.217 | 7.87E-01 | 0.07 |
| **fT4 results** |  |  |  |  |  |  |  |  |
| XXL-VLDL-C | 0.403 | 0.155 | 9.20E-03 | 6.78 | -0.139 | 0.133 | 2.95E-01 | 1.10 |
| XXL-VLDL-CE | 0.422 | 0.158 | 7.64E-03 | 7.12 | -0.126 | 0.133 | 3.46E-01 | 0.89 |
| XXL-VLDL-FC | 0.420 | 0.175 | 1.63E-02 | 5.77 | -0.174 | 0.132 | 1.88E-01 | 1.73 |
| XL-VLDL-C | 0.441 | 0.171 | 9.89E-03 | 6.65 | -0.153 | 0.132 | 2.47E-01 | 1.34 |
| XL-VLDL-CE | 0.455 | 0.175 | 9.44E-03 | 6.74 | -0.157 | 0.132 | 2.35E-01 | 1.41 |
| XL-VLDL-FC | 0.443 | 0.177 | 1.24E-02 | 6.25 | -0.147 | 0.132 | 2.66E-01 | 1.24 |
| IDL-TG | 0.323 | 0.122 | 8.26E-03 | 6.98 | -0.267 | 0.164 | 1.04E-01 | 2.64 |
| L-LDL-TG | 0.291 | 0.123 | 1.82E-02 | 5.58 | -0.302 | 0.185 | 1.03E-01 | 2.65 |
| S-LDL-TG | 0.320 | 0.130 | 1.39E-02 | 6.05 | -0.280 | 0.131 | 3.28E-02 | 4.55 |
| M-HDL-TG | 0.162 | 0.110 | 1.41E-01 | 2.17 | -0.168 | 0.130 | 1.97E-01 | 1.66 |
| S-HDL-PL | -0.187 | 0.072 | 9.03E-03 | 6.82 | -0.216 | 0.135 | 1.10E-01 | 2.56 |
| LDL-TG | 0.430 | 0.178 | 1.59E-02 | 5.82 | -0.186 | 0.130 | 1.53E-01 | 2.04 |
| HDL-TG | 0.344 | 0.072 | 1.55E-06 | 23.08 | -0.260 | 0.133 | 5.12E-02 | 3.80 |
| DAG | 0.075 | 0.077 | 3.30E-01 | 0.95 | -0.078 | 0.138 | 5.73E-01 | 0.32 |
| DAG/TG | -0.031 | 0.078 | 6.93E-01 | 0.16 | -0.029 | 0.140 | 8.37E-01 | 0.04 |
| CLA | 0.015 | 0.182 | 9.35E-01 | 0.01 | -0.091 | 0.138 | 5.11E-01 | 0.43 |
| SFA | 0.353 | 0.151 | 1.91E-02 | 5.49 | -0.395 | 0.132 | 2.84E-03 | 8.91 |
| CLA/FA | -0.068 | 0.185 | 7.12E-01 | 0.14 | -0.086 | 0.139 | 5.37E-01 | 0.38 |
| His | 0.135 | 0.068 | 4.77E-02 | 3.92 | -0.106 | 0.131 | 4.16E-01 | 0.66 |
| Tyr | 0.082 | 0.070 | 2.40E-01 | 1.38 | 0.276 | 0.197 | 1.61E-01 | 1.97 |
| AcAce | -0.111 | 0.072 | 1.23E-01 | 2.37 | -0.044 | 0.314 | 8.89E-01 | 0.02 |

Abbreviations: TSH; thyroid stimulating hormone, fT4; free thyroxine, SE; standard error, N.A.; not available.

Pooled estimates, standard errors and p-values were derived from a random effects model meta-analysis of study-level multivariable regression adjusted for age, sex, body mass index and smoking status.

**Online Table 8. Results for sensitivity analyses for MR on thyroid status and CAD**

|  | **IVW** | | **WME** | | **MR Egger** | | | | **MR-PRESSO** | |
| --- | --- | --- | --- | --- | --- | --- | --- | --- | --- | --- |
|  | **Odds Ratio  (95% CI)** | **P-**  **value** | **Odds Ratio  (95% CI)** | **P-**  **value** | **Odds Ratio  (95% CI)** | **P-**  **value** | **Intercept  (95% CI)** | **P-**  **value** | **Global**  **P-value** | **Distortion**  **P-value** |
| **TSH** |  |  |  |  |  |  |  |  |  |  |
| CARDIoGRAM | 1.09 (0.97;1.21) | 0.13 | 1.07 (0.95;1.21) | 0.27 | 0.96 (0.73;1.26) | 0.78 | 0.01 (-0.01;0.03) | 0.34 | <0.001 | 0.67 |
| UK Biobank | 1.01 (0.96;1.06) | 0.71 | 1.03 (0.98;1.09) | 0.25 | 1.02 (0.91;1.15) | 0.71 | -0.00 (-0.01;0.01) | 0.80 | <0.001 | 0.67 |
| FinnGen | 1.05 (0.97;1.13) | 0.20 | 1.12 (1.01;1.24) | 0.01 | 0.98 (0.81;1.19) | 0.83 | 0.01 (-0.01;0.02) | 0.44 | 0.13 | N.A. |
| Overall | 1.03 (0.99;1.07) | 0.16 | 1.06 (1.00;1.12) | 0.04 | 1.00 (0.91;1.10) | 0.92 | N.A. | N.A. | N.A. | N.A. |
| **fT4** |  |  |  |  |  |  |  |  |  |  |
| CARDIoGRAM | 0.94 (0.81;1.08) | 0.39 | 1.09 (0.90;1.30) | 0.38 | 1.11 (0.77;1.61) | 0.58 | -0.01 (-0.04;0.01) | 0.35 | 0.11 | N.A. |
| UK Biobank | 1.01 (0.97;1.05) | 0.71 | 0.99 (0.92;1.06) | 0.70 | 1.03 (0.93;1.14) | 0.52 | -0.00 (-0.01;0.01) | 0.59 | 0.54 | N.A. |
| FinnGen | 1.01 (0.90;1.13) | 0.90 | 1.01 (0.89;1.16) | 0.85 | 0.90 (0.68;1.17) | 0.43 | 0.01 (-0.01;0.03) | 0.36 | 0.09 | N.A. |
| Overall | 1.00 (0.96;1.04) | 0.89 | 1.00 (0.95;1.05) | 0.98 | 1.02 (0.93;1.12) | 0.65 | N.A. | N.A. | N.A. | N.A. |

Abbreviations: IVW; Inverse-variance weighted, WME; Weighted Median Estimator, MR-PRESSO; Mendelian randomization pleiotropy residual sum and outlier, TSH; thyroid stimulating hormone, fT4; free thyroxine, N.A.; not applicable.

**
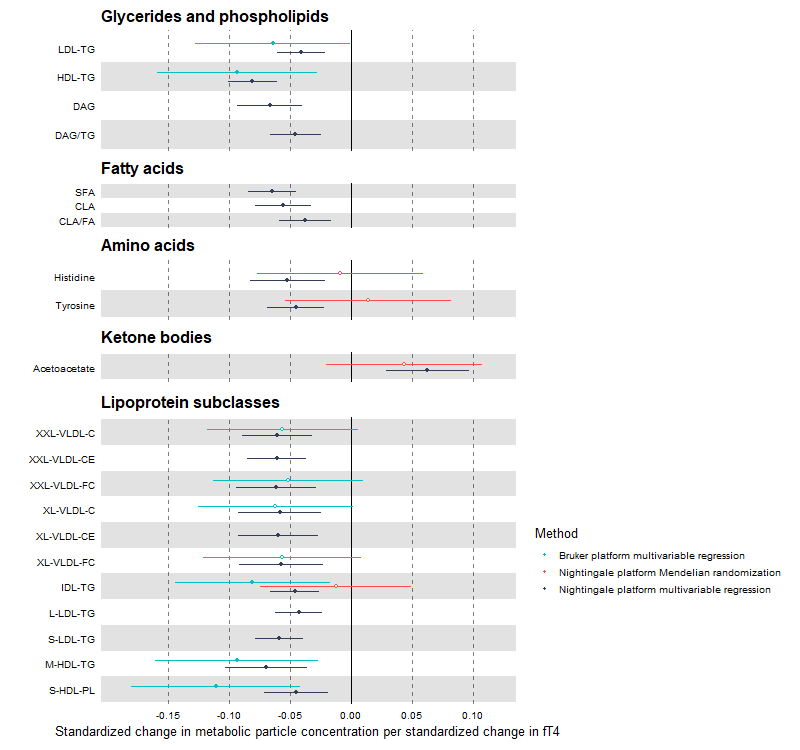
**

**Online Figure 1. Second stage associations between fT4 and 21 metabolomic markers**

Estimates derived from multivariable regression and Mendelian randomization analyses on Nightingale platform and multivariable regression analysis on Bruker metabolomics platform


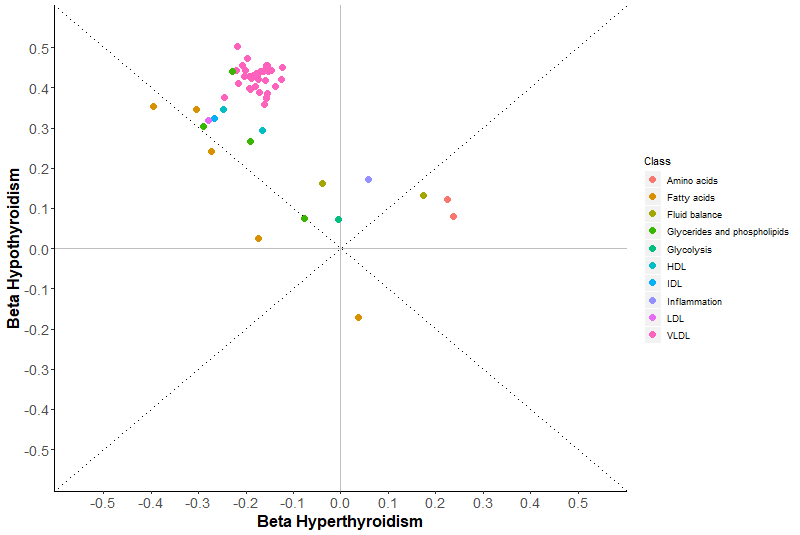

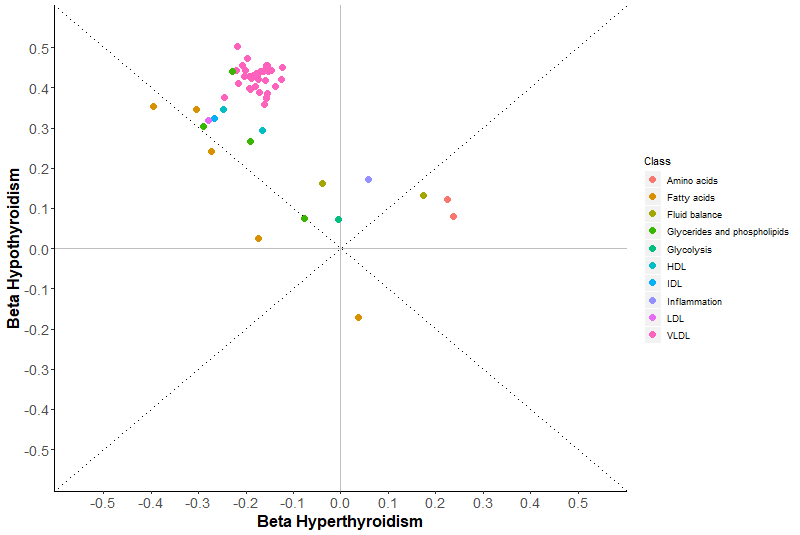
 **A**


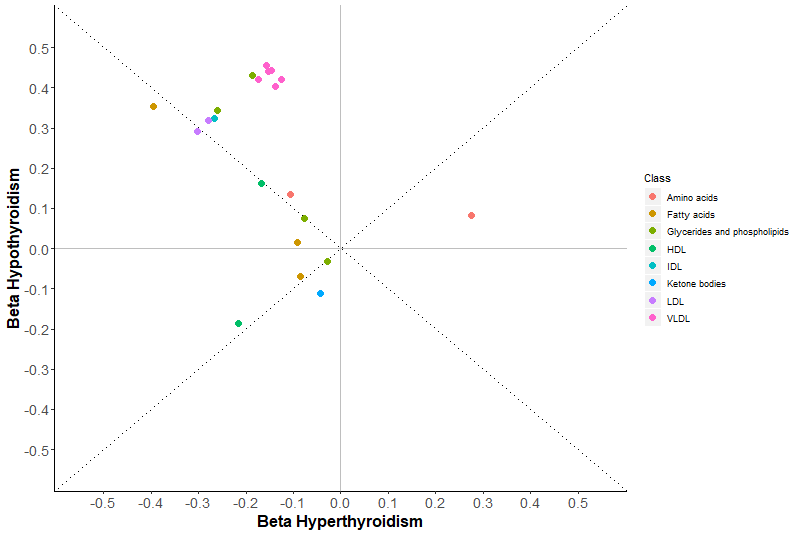

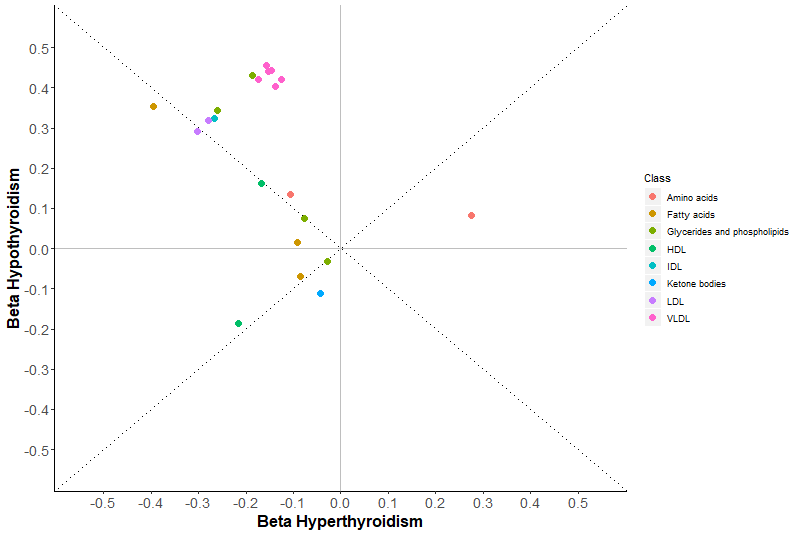
 **B**

**Online Figure 2. Association of thyroid dysfunction with metabolomic markers identified for TSH and fT4 in first stage**

A) Associations between hypo- and hyperthyroidism and 52 metabolomic markers associated with TSH,
B) Associations between hypo- and hyperthyroidism and 21 metabolomic markers associated with fT4
